# Distinct mutational features across preinvasive and invasive subtypes identified through comprehensive profiling of surgically resected lung adenocarcinoma

**DOI:** 10.1038/s41379-022-01076-w

**Published:** 2022-05-31

**Authors:** Chan Xiang, Chunyu Ji, Yiran Cai, Haohua Teng, Yulu Wang, Ruiying Zhao, Zhanxian Shang, Lianying Guo, Shengnan Chen, Analyn Lizaso, Jing Lin, Haozhe Wang, Bing Li, Zhou Zhang, Jikai Zhao, Jinzhi Wei, Jiaxin Liu, Lei Zhu, Wentao Fang, Yuchen Han

**Affiliations:** 1 Department of Pathology, Shanghai Chest Hospital, Shanghai Jiao Tong University, Shanghai, 200030 China; 2 Department of Thoracic Surgery, Shanghai Chest Hospital, Shanghai Jiao Tong University, Shanghai, 200030 China; 3 Burning Rock Biotech, Guangzhou, 510300 China

**Keywords:** Cancer genomics, Non-small-cell lung cancer, Oncogenes

## Abstract

Lung adenocarcinoma (LUAD) is a heterogeneous disease. Our study aimed to understand the unique molecular features of preinvasive to invasive LUAD subtypes. We retrospectively analyzed the clinical, histopathological, and molecular data of 3,254 Chinese patients with preinvasive lesions (*n* = 252), minimally invasive adenocarcinomas (*n* = 479), and invasive LUAD (*n* = 2,523). Molecular data were elucidated using a targeted 68-gene next-generation sequencing panel. Our findings revealed four preinvasive lesion-predominant gene mutations, including *MAP2K1* insertion-deletions (indels), *BRAF* non-V600E kinase mutations, and exon 20 insertions (20ins) in both *EGFR* and *ERBB2*, which we referred to as mutations enriched in AIS (MEA). The detection rate of MEA in invasive tumors was relatively lower. *MAP2K1* missense mutations, which were likely passenger mutations, co-occurred with oncogenic driver mutations, while small indels were mutually exclusive from other genes regardless of the invasion level. *BRAF* non-V600E kinase-mutant invasive adenocarcinomas (IAC) had significantly higher mutation rates in tumor suppressor genes but lower frequency of co-occurring oncogenic driver mutations than non-kinase-mutant IAC, suggesting the potential oncogenic activity of *BRAF* non-V600E kinase mutations albeit weaker than *BRAF* V600E. Moreover, similar to the extremely low frequency of *MAP2K1* indels in IAC, *BRAF* non-V600E kinase domain mutations co-occurring with *TSC1* mutations were exclusively found in preinvasive lesions. Compared with *EGFR* L858R and exon 19 deletion, patients with preinvasive lesions harboring 20ins in either *EGFR* or *ERBB2* were significantly younger, while those with IAC had similar age. Furthermore, our study demonstrated distinct mutational features for subtypes of oncogene mutations favored by different invasion patterns in adenocarcinomas. In conclusion, our data demonstrate distinct mutational features between preinvasive lesions and invasive tumors with MEA, suggesting the involvement of MEA in the early stages of tumorigenesis. Further pre-clinical studies are required to establish the role of these genes in the malignant transformation of LUAD.

## Introduction

Lung adenocarcinoma (LUAD) is a subtype of non-small cell lung cancer (NSCLC) and accounts for approximately 50% of all lung cancer cases diagnosed^1,2^. LUAD is characterized by distinct histological and molecular features. LUAD involves a complex mixture of several growth patterns contributing to its heterogeneity^1–3^. The 2015 World Health Organization (WHO) guidelines have refined the LUAD classification according to their predominant growth pattern and the general pattern of invasion^1–3^. LUAD with predominantly lepidic, non-mucinous pattern is characterized depending on its invasion pattern as preinvasive adenocarcinoma in situ (AIS), minimally invasive adenocarcinoma (MIA), or fully invasive adenocarcinoma (IAC) with lepidic component. In contrast, other invasive non-lepidic LUAD with identifiable morphologic patterns are classified according to their predominant growth patterns^1,2^. Mounting evidence has demonstrated that various predominant histologic subtypes were associated with disease recurrence risk^4,5^. Over the years, tremendous efforts have been invested in elucidating the molecular features of LUAD that have contributed to our current understanding of its molecular heterogeneity^6–15^. These collective efforts have revealed distinct molecular features, as well as potentially actionable mutations, that helped shape the current treatment landscape of LUAD, particularly for IAC^6–8,11,13–17^. In recent years, the use of enhanced radiological techniques in lung cancer diagnosis has contributed to identifying more patients with preinvasive lung nodules in clinical practice. Hence, the molecular landscape of preinvasive LUAD and the molecular mechanisms involved in the transformation from preinvasive to invasive LUAD are just beginning to be understood^10,12–15,18^.

In this study, we aimed to understand the unique molecular features of preinvasive to invasive LUAD subtypes. To achieve this, we retrospectively analyzed the clinical, histopathological, molecular, and radiological data of 3,254 Chinese patients whose surgically-resected specimens were submitted for histopathologic and genomic analysis. We also performed comparative analyses to uncover distinct mutational features between preinvasive and invasive LUADs involved in malignant transformation.

## Materials and methods

### Patient selection

A total of 3254 previously untreated LUAD patients who underwent surgical resection of pulmonary nodules and submitted tumor samples for molecular testing between January 2018 and June 2019 at Shanghai Chest Hospital were included in this study. All cases were pathologically-confirmed preinvasive lesions and stage IA-IIIA LUAD by at least two pathologists. Baseline clinicopathological information was retrieved from the electronic medical record database of Shanghai Chest Hospital, including age, sex, smoking history, radiologic findings, pathological tumor size, pathological TNM stage, visceral pleural invasion status, and histopathologic subtypes. Informed consent was obtained from all subjects, and the study was approved by the Ethics Committee of Shanghai Chest Hospital and conducted in accordance with the Declaration of Helsinki of 1964 and its later amendments.

### Radiological evaluation

All patients underwent preoperative evaluation with chest thin-section computed tomography (CT) scanning. The CT images were reviewed independently by at least two board-certified radiologists with discrepancies resolved through a consensus. Radiologic subgroups were categorized according to CT appearance as pure ground-glass opacities (GGO), mixed GGO with part-solid components, or pure solid nodules.

### Histopathological evaluation and TNM staging

All resected specimens were processed as formalin-fixed paraffin-embedded (FFPE) blocks and stained with hematoxylin-eosin (HE) according to the standard histopathologic procedures. Lung adenocarcinoma subtypes were classified independently by two experienced pathologists according to the 2015 WHO classification of lung tumors as AAH, AIS, MIA, and IAC^1–3^. Histological subtypes of IAC were classified according to the predominant subtype after comprehensive histological subtyping by a semiquantitative estimation of the percentage of all patterns in 5% increments. Any discordant findings between the two pathologists were resolved through review and discussion until consensus was reached. Post-operative pathological staging evaluating the primary lung tumor (T), affected lymph node (N), and metastasis (M) were performed based on the American Joint Committee on Cancer (AJCC) eighth edition of the TNM staging system of NSCLC^2,19^. Pathological tumor size was defined as the maximum diameter on pathological examination of the surgically removed tumor. The visceral pleural invasion status was defined based on the degree of pleural invasion using elastic stain as PL0, PL1, PL2, and PL3^19^. For patients with multiple specimens derived from ≥2 lung lesions, the tumor size, histological subtype, and genomic profile were evaluated using either the main tumor or the largest nodule.

### Targeted next-generation sequencing (NGS) and sequencing analysis

Tissue DNA was extracted from the FFPE sections of surgically-resected tumor tissue using QIAamp DNA FFPE tissue kit (Qiagen, Hilden, Germany). The quality and size of the fragments were assessed using Qubit 2.0 Fluorimeter with the dsDNA high-sensitivity assay kit (Life Technologies, Carlsbad, CA). A minimum of 50 ng of tissue DNA is required for NGS library preparation. Samples having either insufficient DNA quantity or inadequate quality for subsequent NGS procedures were excluded. NGS library was constructed, and target capture was performed according to optimized protocols using a panel consisting of 68 lung cancer-related genes spanning 245 kilobases of the human genome (Lung Core, Burning Rock Biotech, Guangzhou, China). The gene list is tabulated in Table S1. Indexed samples were sequenced on MiseqDx or Nextseq500 (Illumina, Inc., CA, USA) with paired-end reads and target sequencing depth of 1,000×. Sequencing analysis was performed using an optimized bioinformatics pipeline that enables accurate detection of somatic variants by discriminating sequencing artifacts from real mutations as described previously^20^.

Copy number variations (CNVs) were analyzed based on the depth of coverage data of capture intervals. Coverage data were corrected against sequencing bias resulting from GC content and probe design. The average coverage of all captured regions was used to normalize the coverage of different samples to comparable scales. Copy number (CN) was calculated based on the ratio between the depth of coverage in tumor samples and average coverage of an adequate number (n>50) of samples without CNVs as reference per capture interval. CNV is called if the coverage data of the gene region was quantitatively and statistically different from its reference control. The limit of detection for CNV is 1.5 for CN deletion and 2.64 for CN amplifications. The CNV spectrum of a patient was reflected as chromosomal fluctuation coefficient, referred to as varscore, according to a previously described method^21^. In summary, a higher varscore indicates a greater number of CNV a patient harbored, as well as higher chromosomal fluctuation and instability.

### Statistical analysis

All statistical analyses were performed using R software (R v.3.4.0; R: The R-Project for Statistical Computing, Vienna, Austria). The distribution of mutations according to histological subtype was analyzed using either two-sided Fisher’s test or chi-square test. The difference in molecular, histopathological, or radiological features across LUAD subtypes was analyzed using student *t*-test or Wilcoxon signed-rank test. A *p*-value <0.05 was considered statistically significant.

## Results

### Clinical and histopathologic features of the cohort

Our study cohort was comprised of 3254 Chinese patients with resected preinvasive lesions (stage 0) and stage IA-IIIA LUAD. Of them, 60.8% were female, with a median age of 61 years (range 19-87 years). Approximately two-thirds (68.2%) of our cohort were non-smokers; however, a significantly higher propotion of smoking history was shown in patients with IAC than those with either MIA or AIS (21.8% vs. 7.3% vs. 6.5%, *P* < 0.001; Fig. S1A). Tumor size was significantly larger among patients with IAC than patients with either AIS or MIA (*P* < 0.001; Fig. S1B). A majority of the cohort was pathologically diagnosed with stage IA lung cancer (61.1%, *n* = 1,988). Multifocal lung adenocarcinoma was identified in 19.0% (*n* = 617) of our cohort. Table 1 summarizes the baseline demographics and clinicopathologic features of the cohort.

**Table 1:** Baseline clinicopathologic features of the cohort.

	*n* (%)				
Clinicopathological features	Overall (*n* = 3254)	AAH (*n* = 6)	AIS (*n* = 246)	MIA (*n* = 479)	IAC (*n* = 2523)
Age (median [IQR]; years)	61.0 [52.0, 66.0]	50.5 [48.5, 59.3]	51.0 [43.0, 59.0]	54.0 [44.0, 63.0]	62.0 [55.0, 67.0]
Sex					
Female	1979 (60.8)	2 (33.3)	187 (76.0)	349 (72.9)	1441 (57.1)
Male	1275 (39.2)	4 (66.7)	59 (24.0)	130 (27.1)	1082 (42.9)
Smoking history/status					
Non-smoker	2218 (68.2)	3 (50.0)	213 (86.6)	410 (85.6)	1592 (63.1)
Smoker	603 (18.5)	2 (33.3)	16 (6.5)	35 (7.3)	550 (21.8)
Data not available	433 (13.3)	1 (16.7)	17 (6.9)	34 (7.1)	381 (15.1)
Radiological type of pulmonary nodule					
Pure GGO	549 (16.9)	5 (83.3)	210 (85.4)	254 (53.0)	80 (3.2)
Mixed GGO	1429 (43.9)	1 (16.7)	33 (13.4)	209 (43.6)	1186 (47.0)
Solid nodule	1247 (38.3)	0 (0.0)	3 (1.2)	14 (2.9)	1230 (48.8)
Data not available	29 (0.9)	0 (0.0)	0 (0.0)	2 (0.4)	27 (1.1)
Tumor size (median [IQR]; cm)	1.8 [1.1, 2.6]	0.5 [0.3,0.7]	0.7 [0.6,0.9]	0.9 [0.7, 1.2]	2.1 [1.5, 3.0]
Visceral pleural invasion status					
PL0	2713 (83.4)	6 (100.0)	246 (100.0)	479 (100.0)	1982 (78.6)
PL1	363 (11.2)	0 (0.0)	0 (0.0)	0 (0.0)	363 (14.4)
PL2	156 (4.8)	0 (0.0)	0 (0.0)	0 (0.0)	156 (6.2)
PL3	8 (0.2)	0 (0.0)	0 (0.0)	0 (0.0)	8 (0.3)
Data not available	14 (0.4)	0 (0.0)	0 (0.0)	0 (0.0)	14 (0.6)
Pathological stage					
NA (AAH)	6 (0.2)	6 (100.0)	0 (0.0)	0 (0.0)	0 (0.0)
0	246 (7.6)	0 (0.0)	246 (100.0)	0 (0.0)	0 (0.0)
IA1	643 (19.8)	0 (0.0)	0 (0.0)	479 (100.0)	164 (6.5)
IA2	917 (28.2)	0 (0.0)	0 (0.0)	0 (0.0)	917 (36.3)
IA3	428 (13.2)	0 (0.0)	0 (0.0)	0 (0.0)	428 (17.0)
IB	448 (13.8)	0 (0.0)	0 (0.0)	0 (0.0)	448 (17.8)
IIA	74 (2.3)	0 (0.0)	0 (0.0)	0 (0.0)	74 (2.9)
IIB	157 (4.8)	0 (0.0)	0 (0.0)	0 (0.0)	157 (6.2)
IIIA	335 (10.3)	0 (0.0)	0 (0.0)	0 (0.0)	335 (13.3)
Multifocal lung adenocarcinoma status					
No	2637 (81.0)				
Yes	617 (19.0)				

*AAH*, atypical adenomatous hyperplasia; *AIS*, adenocarcinoma in situ; *IQR*, interquartile range; *GGO*, ground-glass opacity; *IAC*, invasive adenocarcinoma; *MIA*, minimally invasive adenocarcinoma; *NA* denotes clinical stage not applicable; *PL*, pleura.

**Fig. 1:**
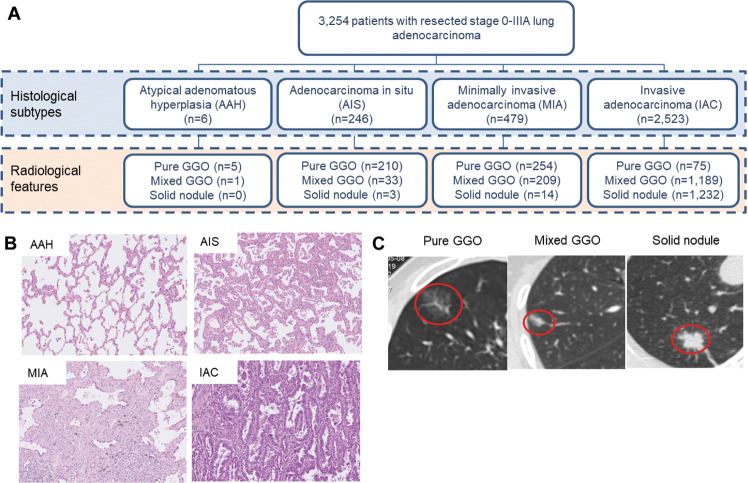
Study overview. **A** Flow diagram of patient distribution according to histological subtypes and radiological features. **B** Representative hematoxylin-eosin (HE)-stained images for the histological features of atypical adenomatous hyperplasia (AAH), adenocarcinoma in situ (AIS), minimally invasive adenocarcinoma (MIA), and invasive adenocarcinoma (IAC). **C** Computed tomography images of the pulmonary nodules illustrating the pure ground-glass opacity (GGO), mixed GGO, and solid nodule. Nodules were encircled in red.

Figure 1 illustrates the distribution of the cohort according to general histological subtypes and radiological features of the pulmonary nodules. According to histological subtypes, the cohort was comprised of 0.2% atypical adenomatous hyperplasia (AAH, *n* = 6), 7.6% AIS (*n* = 246), 14.7% MIA (*n* = 479), and 77.5% IAC (*n* = 2523), including 42.0% acinar (*n* = 1367), 12.7% papillary (*n* = 412), 9.3% lepidic (*n* = 301), 7.5% solid (*n* = 243), 2.9% micropapillary (*n* = 93), 2.6% invasive mucinous (*n* = 85), 0.6% enteric (*n* = 18), 0.1% fetal (*n* = 3), and 0.03% colloid (*n* = 1) predominant subtypes (Table S2). Table S2 summarizes the clinical, histopathological, radiological, and molecular characteristics of the cohort according to LUAD histological subtypes.

### Mutational features of the cohort

Analysis of the genomic profiles of the surgically-resected tissue samples from our cohort identified a total of 9133 somatic mutations in 64 genes from 3224 patients, resulting in a mutation detection rate of 99.1% (Fig. S2A). Of these, 3,019 patients were detected with actionable mutations in any of the eight classic NSCLC oncogenic drivers, including *EGFR*, *ALK*, *BRAF*, *ERBB2*, *KRAS*, *MET*, *RET*, and *ROS1*. *EGFR* was the most commonly mutated gene, detected in 66.3% (*n* = 2157) of the cohort (Fig. S2B). *TP53* was the most common concurrent mutation detected in 29.0% of the cohort (*n* = 944, Fig. S2B). A majority (72.3%) of the mutations identified were missense mutations (55.8%, *n* = 4,455) and insertion-deletion mutations (indels) (16.5%, *n* = 1320) (Fig. S2C).

### Mutational features of three histological subtypes according to the general invasion pattern

**Fig. 2:**
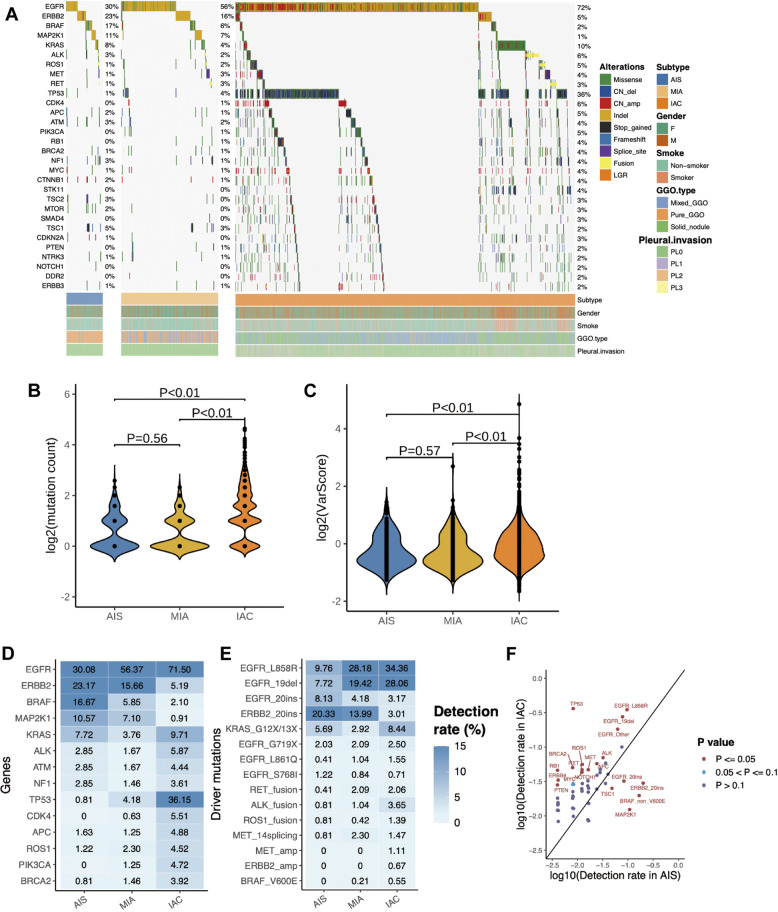
Distinct somatic mutation profiles of preinvasive and invasive LUAD. **A** Oncoprint summarizing the mutational landscape of adenocarcinoma in situ (AIS), minimally invasive adenocarcinoma (MIA), and invasive adenocarcinoma (IAC). The histological subtypes (AIS/MIA/IAC), gender (Male/Female), smoking status (Smoker/Non-smoker), radiological phenotypes (ground-glass opacity (GGO) type; Mixed/Pure/Solid), and visceral pleural invasion status of each patient are indicated by various colors at the bottom of the oncoprint. Each column represents a patient and each row represents a gene. Values on the right represent the percentage of patients with mutations of a specific gene indicated on the left. Different colors denote the mutation types. Violin plots showing the significantly different mutation counts (**B**) and copy number variation (CNV) varscore (**C**) in AIS, MIA, and IAC. Heat map demonstrating the differential mutation detection rates in various genes (**D**) and oncogenic driver genes (**E**) across AIS, MIA, and IAC. **F** Dot plot illustrating the gene mutations that are significantly different between AIS and IAC. Red dots indicate a significantly higher detection rate (*P* value ≤0.05). False discovery rate corrected *P*-values were listed in Table S3.

We further analyzed the mutational features of preinvasive lesions, minimally-invasive lesions, and fully invasive LUAD, which revealed a unique mutation profile among AIS, MIA, and IAC (Fig. 2A). Comparative analyses demonstrated that mutation counts in AIS and MIA were statistically similar (*P* = 0.56), while IAC had a significantly higher mutation counts compared to AIS (*P* < 0.01) and MIA (*P* < 0.01) (Fig. 2B). The distribution of mutation types, particularly missense mutations, small indels, and copy number (CN) amplifications, was distinct across AIS, MIA, and IAC (Fig. S3A). The proportion of indels was significantly higher in AIS than in IAC (36.6% vs. 14.0%; *P* < 0.01), whereas the proportion of CN amplifications was significantly higher in IAC than in AIS and MIA (10.8% vs. 2.8% vs. 1.5%; *P* < 0.01) (Fig. S3A). A majority of CN amplifications were only detected in IAC and not detected in AAH, AIS, or MIA (Fig. S3B). Consistently, chromosomal fluctuation coefficient or CNV varscore was significantly higher in IAC compared to AIS (*P*<0.01) and MIA (*P* < 0.01), whereas CNV varscore was not statistically different between AIS and MIA (*P* = 0.57) (Fig. 2C). These data suggest that the higher mutation counts and genomic instability resulting from an increased number of CNVs could potentially contribute to a more aggressive invasion pattern and could drive the invasive transformation of preinvasive lesions.

Further analyses across AIS, MIA, and IAC revealed that some gene mutations were directly proportional or inversely proportional to the general invasion pattern. AIS were commonly detected with mutations in *EGFR* (30.1%), *ERBB2* (23.2%), *BRAF* (16.7%), and *MAP2K1* (10.6%) (Fig. 2D). The mutation rates in *ERBB2* (23.2% vs. 15.7% vs. 5.2%; *P* < 0.01), *BRAF* (16.7% vs. 5.9% vs. 2.1%; *P* < 0.01), and *MAP2K1* (10.6% vs. 7.1% vs. 0.9%; *P* < 0.01) were inversely proportional with the general invasion pattern, with the highest rates found in AIS followed by MIA, and the lowest rates found in IAC (Fig. 2D). Mutations in *ERBB2*, particularly exon 20 insertions (20ins), were more predominant in AIS (*P* < 0.01) and had mutation rates of 20.3% for AIS, 14.0% for MIA, and 3.0% for IAC (Fig. 2E). Compared to AIS and MIA, IAC harbored significantly higher mutation rates in *TP53* (36.1% vs. 0.8% vs. 4.2%; *P* < 0.01; Fig. 2D) and *EGFR* (71.5% vs. 30.1% vs. 56.4%; *P* < 0.01; Fig. 2D). The two major *EGFR* alterations in IAC were *EGFR* L858R (34.4% vs. 9.8% vs. 28.2%; *P* < 0.01; Fig. 2E) and exon 19 deletion (19del; 28.1% vs. 7.7% vs. 19.4%; *P* < 0.01; Fig. 2E), whereas *EGFR* 20ins was the most predominant *EGFR* alteration in AIS with a mutation rate of 8.1% compared to 4.2% in MIA, and 3.2% in IAC (Fig. 2E).

Comparative analysis revealed that AIS harbored significantly more *BRAF* non-V600E, *EGFR* 20ins, *ERBB2* 20ins, *MAP2K1*, and *TSC1* mutations than IAC (false discovery rate-corrected *P* < 0.05; Fig. 2F; Table S3). The co-occurrences and mutual exclusivity analysis of the five mutations mentioned above across invasion patterns revealed that *TSC1* mutations were significantly associated with *BRAF* non-V600E in AIS and MIA (*P* < 0.01, Fig. S4A, B), while this relationship was not observed in IAC (Fig. S4C). Meanwhile, the other four mutations were mutually exclusive from each other in AIS and MIA (Fig. S4A, B). In IAC, *EGFR* 20ins and *ERBB2* 20ins were more likely to be mutually exclusive from oncogenes such as *KRAS* and *ALK*, whereas *MAP2K1* and *BRAF* non-V600E mutations were more likely to co-occur with *STK11*, *TP53*, *RET*, and *MET* (Fig. S4C). These findings revealed the diversity in co-occurring mutations for preinvasive lesions harboring the four mutations, including *BRAF* non-V600E located in the kinase domain, *EGFR* 20ins, *ERBB2* 20ins, and *MAP2K1* indels, which we further referred to as mutations enriched in AIS (MEA). This diverse landscape of co-occurring mutations in MEA-mutant AIS could facilitate the identification of key mutations associated with preinvasive lesions and help to understand their roles during tumorigenesis.

### Pathway analysis

To further understand the critical pathways involved in the transformation of preinvasive lesions to invasive tumors, we further analyzed the various gene mutations and mapped their relationship to certain networks and signaling pathways (Fig. S5). Pathway analysis revealed that mutations in genes involved in the ERBB pathway were similarly high among AIS, MIA, and IAC (55% vs. 73% vs. 77%), suggesting the key involvement of the ERBB pathway throughout lung tumor development and progression. In addition, mutations in genes involved in the mitogen-activated protein kinase (MAPK) pathway were significantly higher in AIS compared to MIA and IAC (27% vs. 13% vs. 4%; *P* < 0.01), suggesting the critical role of the MAPK pathway in preinvasive stages of tumor development. Contrastingly, mutations in genes involved in the p53 pathway were significantly higher in IAC compared to AIS and MIA (40% vs. 5% vs. 6%; *P* < 0.01), indicating the role of the p53 pathway in tumor invasion and malignant progression.

### Distinct mutational features for *MAP2K1* and *BRAF* in preinvasive and invasive LUAD

**Fig. 3:**
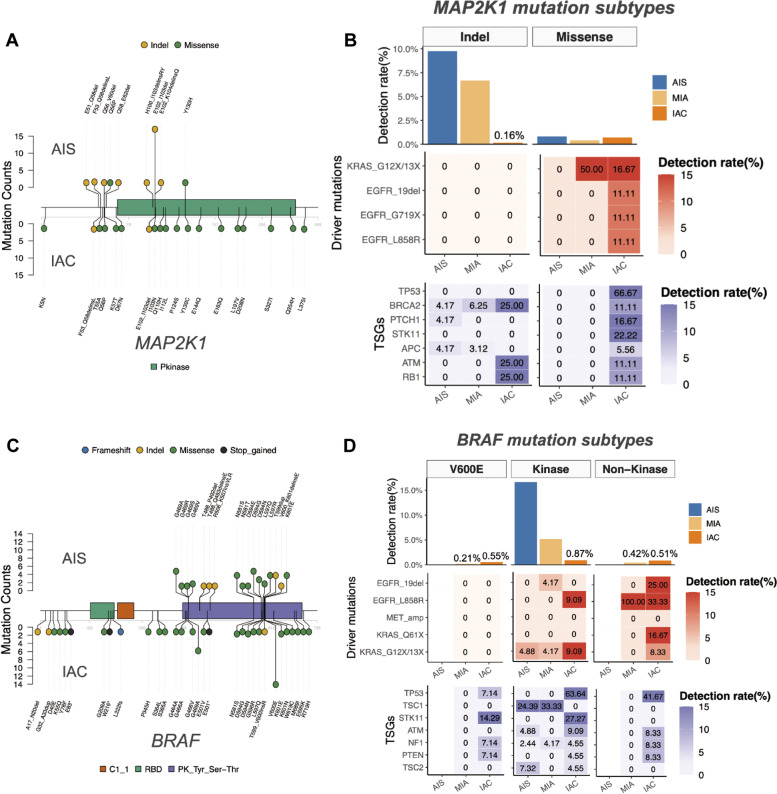
Distinct distribution of *MAP2K1* and *BRAF* mutations in AIS and IAC. AIS tumors harbored more *MAP2K1* insertion-deletion (indel) mutations and *BRAF* non-V600E mutations. Lollipop plots summarizing the mutation sites and mutation types in *MAP2K1* (**A**) and *BRAF* (**C**) detected from the AIS (top) and IAC (bottom). Colors represent the mutation types. Each dot denotes a mutation in the specific site. The height of the lollipop indicates mutation counts. *MAP2K1*-mutant and *BRAF* non-V600E-mutant IAC had distinct concurrent mutations compared to their AIS counterpart. Bar plots at the top illustrate the distribution of detection rate for each mutation subtypes for *MAP2K1* (*i.e*. Indels and missense mutations) (**B**) and *BRAF* (*i.e*. V600E, non-V600E kinase, and non-kinase mutations) (**D**) across AIS, MIA, and IAC. Heat maps at the middle and bottom summarize the detection rate of mutations in oncogenic drivers and tumor suppressor genes (TSGs) of the patients with AIS, MIA, and IAC who harbored certain mutation subtypes of *MAP2K1* (**B**) and *BRAF* (**D**). *BRAF* V600E and non-kinase mutations were not detected in AIS.

In AIS, *MAP2K1* mutations were predominantly small indels, particularly E102_I103del located in the protein kinase domain, with a detection rate of 6.9% (17/246; Fig. 3A). *MAP2K1* E102_I103del was also frequent in MIA (5.4%, Fig. S6A), but significantly less in IAC (<0.01%; *P* < 0.01; Fig. 3A). Among the *MAP2K1*-mutant tumors, indels accounted for 92.3% (24/26) of *MAP2K1* mutations in AIS, 94.1% (32/34) in MIA, and 18.2% (4/22) in IAC. Meanwhile, missense mutations accounted for 7.7% (2/26) of *MAP2K1* mutations in AIS, 5.9% (2/34) in MIA, and 81.8% (18/22) in IAC (Fig. 3A, Fig. S6A, Table S2). All the *MAP2K1* mutations detected from our cohort were listed in Table S4.

In contrast to the predominant detection of oncogenic driver mutations in tumors harboring *MAP2K1* missense mutations (13/22, 59.1%), none of these driver mutations were detected in tumors harboring *MAP2K1* indels (Fig. 3B). Consistently, tumor suppressor gene (TSG) mutations were significantly more frequent in IAC with *MAP2K1* missense mutations (Fig. 3B). Further comparative analysis demonstrated that IAC with *MAP2K1* indels had significantly lower mutation counts but higher relative allele frequency than IAC with other *MAP2K1* mutations (Fig. S6B), suggesting that indels were likely to be clonal oncogenic drivers of tumorigenesis, whereas missense mutations were likely to be passenger mutations.

We further categorized the *MAP2K1* indels and missense mutations detected from our cohort according to their clinical significance as oncogenic/likely oncogenic (O/LO) and variants of unknown significance (VUS) using the OncoKB database^22^. The *MAP2K1* indels were predominantly O/LO (93.3%, 56/60; Fig. S7A). Moreover, the *MAP2K1* indels and missense mutations detected across the histological subtypes were predominantly O/LO mutations (Fig. S7B). *MAP2K1* indels and missense mutations detected in AIS had no concurrent loss-of-function (LOF) mutations in TSGs (Fig. S7C, D). Concurrent LOF mutations in TSGs were only detected in IAC harboring *MAP2K1* indels and missense mutations (Fig. S7C, D). These findings were consistent with the data shown in Fig. 3B.

Among the *BRAF* mutations, non-V600E mutations localized within the protein kinase domain were more predominant in AIS (16.7%; Fig. 3C) and MIA (5.0%; Fig. S6C), while V600E was not detected in AIS (Fig. 3C, D) and only detected in one patient with MIA (Fig. S6C). Contrastingly, V600E was the hotspot *BRAF* mutation among IAC with a mutation rate of 0.6%, while non-V600E mutations, with a collective mutation rate of 1.5%, were more spread out (Fig. 3C). In our cohort, we detected a total of 50 unique *BRAF* mutations across histological subtypes. Table S5 lists all *BRAF* mutations detected in our cohort. For further analysis, the *BRAF* mutations were grouped into three distinct subtypes according to mutation sites, including V600E and non-V600E mutations located in the kinase domain (kinase), and other mutations located outside of the kinase domain (non-kinase). According to this *BRAF* mutation subgrouping, the 50 unique mutations were grouped as follows: *BRAF* V600E was a subgroup, 34 were non-V600E kinase mutations, and 15 were non-kinase mutations. We also categorized the *BRAF* mutations according to their signaling mechanism and kinase activity as class I, II, and III as reported previously^23,24^. The 50 unique mutations were grouped as follows: V600E was under class I, 12 mutations were categorized as class II, 10 mutations as class III, and 27 in unknown class. Of the 34 non-V600E kinase mutations, 12 mutations were class II, 10 mutations were class III, and 12 were under the unknown *BRAF* class. It should be noted that all class II mutations were located in the kinase domain and included in the non-V600E kinase mutations subgroup. All the 15 non-kinase mutations were under the unknown *BRAF* class. Fig. S8A shows the distribution of the cohort according to our *BRAF* mutation subgrouping, 15 patients with V600E were under *BRAF* class I, 91 patients had non-V600E kinase mutations, and 15 patients with non-kinase mutations were under unknown *BRAF* class. Of the 91 patients with non-V600E kinase mutations, 50.5% (*n* = 46) had class II, 36.3% (*n* = 33) had class III, and 13.2% (*n* = 12) had unknown class. Fig. S8B, C show the distribution of the *BRAF* mutation class across AIS, MIA, and IAC. Class I *BRAF* mutations were detected in 0 AIS, 1 patient with MIA, and 14 patients with IAC. Class II *BRAF* mutations were detected in 1 patient with AAH, 24 patients with AIS, 10 patients with MIA, and 11 patients with IAC. Class III mutations were detected in 14 patients with AIS, 11 patients with MIA, and 8 patients with IAC. More than half (58.5%, 24/41) of *BRAF* mutations detected in AIS were class II (Fig. S8B).

In IAC, oncogenic mutations were not detected in *BRAF* V600E-mutant tumors, and demonstrated significant lower co-occurrence in kinase-mutant tumors compared to non-kinase-mutant tumors (22.7% vs. 91.7%, *P* = 0.036, Fig. 3D; Fig. S8D). The trend towards decreased relative allele frequency (Kruskal-Wallis *P* = 0.015) but increased mutation counts (Kruskal-Wallis *P* < 0.001) were also observed across V600E, kinase, and non-kinase-mutant tumors (Fig. S6D). Moreover, *BRAF* non-V600E kinase-mutant and non-kinase mutant IAC were more likely to harbor concurrent mutations in TSGs, including LOF mutations in *TP53*, compared to *BRAF* V600E-mutant IAC (Fig. 3D; Fig. S8D). Taken together, these results demonstrated that *BRAF* non-V600E mutations located in the kinase domain were likely to co-occur with mutations in TSGs and might exhibit weaker oncogenicity compared to V600E. In contrast, other non-V600E mutations located outside of the kinase domain were likely to be passenger mutations.

*BRAF* non-V600E kinase-mutant AIS and MIA were detected with concurrent mutations in TSGs (Fig. 3D). Consistent with Fig. S4A–C, *TSC1* mutations were detected in 24.4% (10/41) of *BRAF* non-V600E kinase-mutant AIS and 33.3% (8/24) of *BRAF* non-V600E kinase-mutant MIA, but none in IAC, suggesting a preinvasive-specific *BRAF* non-V600E kinase and *TSC1* co-mutant subtype among *BRAF*-mutant tumors. Moreover, almost all of *TSC1* mutations (94.4%, 17/18) detected in *BRAF* non-V600E kinase-*TSC1* co-mutant AIS and MIA were identified as LOF mutations, including splice site, frameshift, and stop-gained mutations, whereas *TSC1* LOF mutations were significantly fewer in other *TSC1*-mutant tumors (29.1%, 16/55, *P* < 0.01). In addition, the lower rate of co-occurring mutations (11.1%, 2/18) among *BRAF* non-V600E kinase-*TSC1* co-mutant AIS and MIA were suggestive of features of being oncogenic drivers.

### Distinct molecular features for *EGFR* and *ERBB2* in preinvasive and invasive LUAD

**Fig. 4:**
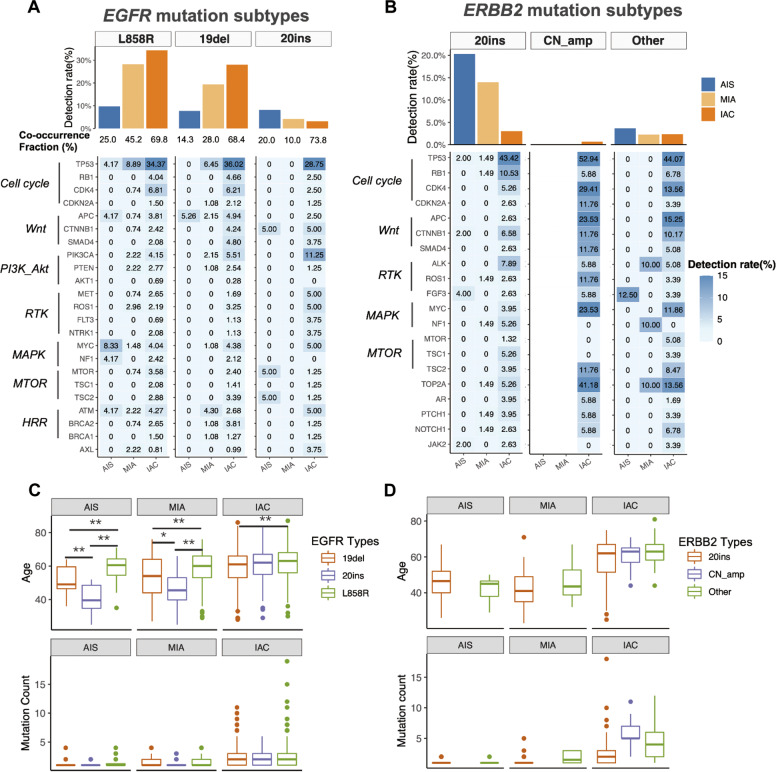
Distinct features of tumors with *EGFR* or *ERBB2* mutation subtypes. *EGFR*-mutant and *ERBB2*-mutant IAC had distinct concurrent mutations compared to their AIS counterpart. Bar plots at the top illustrate the distribution of detection rate for each mutation subtypes for *EGFR* (*i.e*. L858R, exon 19 deletion (19del), and exon 20 insertion (20ins)) (**A**) and *ERBB2* (*i.e*. 20ins, copy number amplification (CN amp), and other non-20ins, non-CNamp mutations) (**B**) across AIS, MIA, and IAC. The co-occurrence fraction below panel A denotes the percentage of co-occurring mutations for each mutation subtype. Heat maps at the bottom summarize the mutations in genes involved in key signaling pathways of the patients with AIS, MIA, and IAC who harbored certain mutation subtypes of *EGFR* (**A**) and *ERBB2* (**B**). Patients harboring certain *EGFR* (**C**) or *ERBB2* (**D**) mutation subtypes had different age of onset (top) and had distinct mutation counts (bottom) across tumor invasion levels.

Comparative analyses demonstrated that AIS had similar frequencies of *EGFR* L858R, 19del, and 20ins (9.8% vs. 7.7% vs. 8.1%, *P* = 0.762), whereas MIA and IAC had statistically more predominant *EGFR* L858R and 19del than *EGFR* 20ins (MIA, 28.2% vs. 19.4% vs. 4.2%, *P* < 0.001; IAC, 34.4% vs. 28.1% vs. 3.2%, *P* < 0.001), suggesting the distinct patterns of *EGFR* driver mutations across tumors of varying invasion levels (Fig. 4A). The two most prevalent mutation subtypes of *EGFR* 20ins in our cohort were p.A767_V769dup (18.3%, 22/120) and p.S768_D770dup (30.0%, 36/120). These two mutation subtypes account for approximately half of *EGFR* 20ins across AIS (55.0%, 11/20), MIA (50.0%, 10/20), and IAC (46.3%, 37/80). In AIS, the proportion of tumors detected with various co-occurring mutations, or co-occurrence fraction, were similar for *EGFR* 20ins, 19del, and L858R (20.0% vs. 14.3% vs. 25.0%, *P* = 0.802; Fig. 4A). Consistently, the co-occurrence fraction was also statistically similar across *EGFR* driver subtypes in IAC (73.8% vs. 68.4% vs. 69.8%, *P* = 0.586). However, the co-occurrence fraction for *EGFR* 20ins-mutant MIA was significantly lower than *EGFR* 19del or L858R-mutant MIA (10.0% vs. 28.0% vs. 45.2%, *P* < 0.001), suggesting that *EGFR*-mutant MIA and IAC had distinct concurrent mutational patterns regardless of the similarly higher prevalence of *EGFR* L858R and 19del than 20ins.

Subsequent analysis of concurrent mutations and their corresponding pathways revealed that cell cycle, Wnt, phosphatidylinositol 3-kinase (PI3K)-Akt, and receptor tyrosine kinase (RTK) signaling pathways were most frequently mutated among *EGFR* driver-mutant IAC (Fig. 4A). Further pairwise comparative analyses demonstrated that *EGFR* 20ins-mutant IAC had significantly higher concurrent oncogenic mutations in *PIK3CA* (11.25% vs. 4.80% vs. 3.00%, *P* < 0.01) compared to *EGFR* 19del-mutant or L858R-mutant IAC (Fig. S9A).

Among *ERBB2*-mutant tumors, 20ins were most frequently identified and showed a reverse pattern by invasion levels with the highest mutation rate in AIS and lowest in IAC (20.3% vs. 14.0 % vs. 3.0%; Fig. 4B). The most prevalent mutation subtype of *ERBB2* 20ins from our cohort was p.A775_G776insYVMA (71.0%, 137/193), which had comparable detection rate across AIS (68.0%, 34/50), MIA (76.1%, 51/67), and IAC (68.4%, 52/76). CN amplifications were exclusively detected in IAC, whereas single-nucleotide variations (SNV) or other mutations were identified with similar detection rate regardless of invasion patterns (3.7% vs. 2.3% vs. 2.4%; Fig. 4B). Similar with *EGFR* driver-mutant IAC, cell cycle, Wnt, RTK, and MAPK signaling pathways were frequently altered among *ERBB2*-mutant IAC (Fig. 4B; Fig. S9B). Concurrent LOF mutations in TSGs were almost undetected among *ERBB2*-mutant AIS and MIA (Fig. S9B).

Investigation of clinical and mutational characteristics revealed that patients with *EGFR* 20ins-mutant AIS and MIA were significantly younger than those with *EGFR* 19del and L858R, whereas this age difference was not observed among *EGFR*-mutant IAC (Fig. 4C). Consistent with their biological oncogenic functions, mutation counts were similarly low among the three *EGFR* driver subtypes and *ERBB2* 20ins-mutant tumors (Fig. 4C, Fig. 4D). Despite the similar age between patients with AIS and MIA harboring *ERBB2* 20ins and those with *ERBB2* SNVs (Fig. 4D), patients with AIS and MIA harboring *ERBB2* 20ins were significantly younger than those harboring other mutations, including *BRAF* kinase mutations and *MAP2K1* indels (Fig. S10A, *P* < 0.001). Comparative analysis among any of the four MEA-mutant tumors and non- MEA-mutant tumors demonstrated that *BRAF* non-V600E kinase-mutant IAC had a significantly higher mutation counts than others in IAC (Fig. S10B, *P* < 0.01). No statistical difference was observed for tumor size and smoking history across MEA-mutant AIS and IAC (Fig. S10C, D).

These data suggest that although *EGFR* 19del, L858R, and 20ins are driver mutations in lung adenocarcinoma, *EGFR* 20ins were more frequently altered in preinvasive AIS, whereas *EGFR* 19del and L858R were frequently altered in invasive tumors. The younger age of patients with either *EGFR* or *ERBB2* 20ins-mutant AIS and MIA, and the diverse co-occurring mutation profiles are both suggestive of distinct oncogenic biological processes of these two gene mutations.

### Mutational features of IAC subtypes

**Fig. 5:**
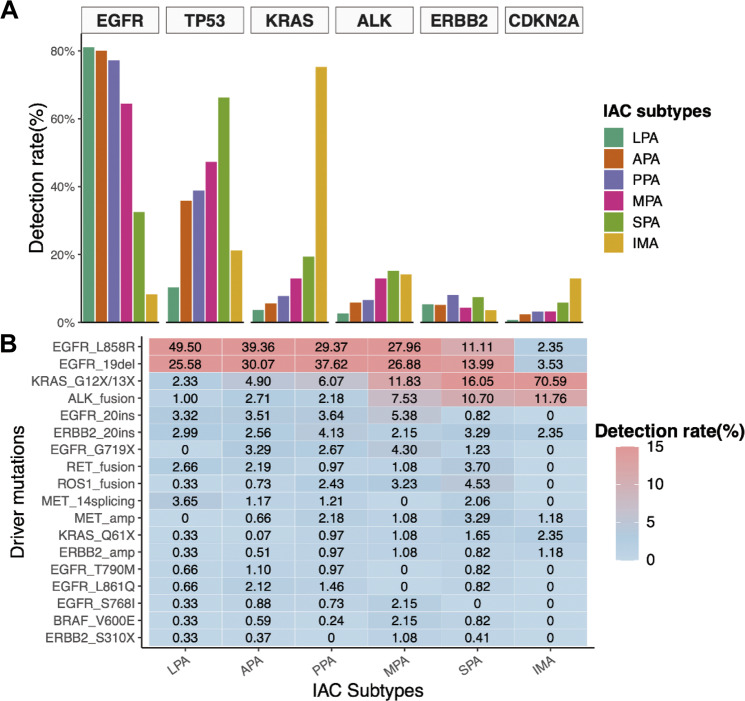
Distinct mutation rates in invasive LUAD subtypes. **A** The distinct mutation rates of the top six genes in various invasive LUAD subtypes including lepidic-predominant adenocarcinoma (LPA), acinar-predominant adenocarcinoma (APA), papillary-predominant adenocarcinoma (PPA), micropapillary-predominant adenocarcinoma (MPA), solid-predominant adenocarcinoma (SPA), and invasive mucinous adenocarcinoma (IMA). **B** Heat map illustrating the differential rates of the activating mutations involving the eight classic NSCLC oncogenes across the six major predominant invasive adenocarcinoma subtypes. The patients with fetal-, colloid-, and enteric-predominant subtypes were not included due to their limited sample size.

Since the predominant growth patterns for IAC are heterogeneous, we further sought to understand the molecular heterogeneity of various invasive growth patterns. Some of the growth patterns, including enteric-, colloid-, and fetal-predominant adenocarcinoma have a very limited sample size and hence were not included in the subsequent analysis. Fig. S11 summarizes the heterogeneous mutational landscape of various IAC subtypes. *EGFR* mutation rates were highest among the subtypes composed predominantly of lepidic (80.1%), acinar (80.1%), papillary (77.2%), and micropapillary (64.5%) patterns (Fig. 5A). The distribution of activating mutations in the eight classic NSCLC oncogenes was also heterogeneous among the IAC subtypes (Fig. 5B). *EGFR* driver mutations, including 19del and L858R, were also uniquely distributed. Papillary-predominant subtype had the highest mutation rate for *EGFR* 19del (37.6%), while the lepidic-predominant subtype had the highest *EGFR* L858R (49.5%; Fig. 5B). *ALK* rearrangements were highest in invasive mucinous (11.8%), solid (10.7%), and micropapillary (7.5%) predominant subtypes (Fig. 5B). *ROS1* and *RET* rearrangements were also highest in the solid-predominant subtype (Fig. 5B). Among the IAC subtypes, invasive mucinous adenocarcinoma had significantly higher mutation rates in *CDKN2A* (12.9%, *P* < 0.01), and *KRAS (*75.3%, *P* < 0.01), particularly *KRAS* G12X (68.2%; *P* < 0.01), *KRAS* G13X (2.3%), and *KRAS* Q61X (2.3%), while having the lowest *EGFR* mutation rate (8.2%, *P* < 0.01), particularly *EGFR* sensitizing mutations 19del (3.5%) and L858R (2.3%; Fig. 5A, B). Meanwhile, solid-predominant adenocarcinoma had the highest *TP53* mutation rate (66.3%, *P* < 0.01; Fig. 5A). The distribution of MEA was similar across the IAC subtypes (*P* = 0.58; Fig. S11).

### Molecular features according to radiological characteristics of pulmonary nodules

We further analyzed the relationship between mutation profile and radiological feature of the pulmonary nodule across various LUAD histological subtypes stratified according to tumor invasiveness. Of the 3254 patients in the cohort, 16.7% (*n* = 544) had pure ground-glass opacities (GGO) with no solid components, 44.0% (*n* = 1432) had subsolid pulmonary nodules composed of both GGO and solid components, also termed as mixed GGOs, and 38.4% (*n* = 1249) had pure solid pulmonary nodules. The remaining 29 patients did not have available data on the CT characteristics of their pulmonary nodules (Table 1). Pulmonary nodules that are composed of either pure (*P* < 0.01) or mixed (*P* < 0.01) GGOs had significantly lower overall mutation detection rates than solid pulmonary nodules (Fig. S12A). Meanwhile, solid nodules had significantly higher CNV detection rates, particularly in *EGFR*, *CDK4*, and *MYC* (Fig. S12B). Moreover, CNV varscore was also significantly higher in solid nodules than in pure GGOs (*P* < 0.01) or mixed GGOs (*P* < 0.01; Fig. S12C).

Consistent with the overall mutation rate of the cohort (Fig. S2C), mutation rates of patients who harbored pure GGOs (Fig. S13A) or mixed GGOs (Fig. S13B) were similar when analyzed according to the general invasion pattern of LUAD subtypes. Among the patients with pure GGOs (Fig. S13A) or mixed GGOs (Fig. S13B), those with AIS harbored the least number of *EGFR* mutations, but a greater number of *ERBB2*, *BRAF*, and *MAP2K1* mutations. Meanwhile, those with IAC had the highest *EGFR* and *TP53* mutation rates and the lowest mutation rates in *ERBB2*, *BRAF*, and *MAP2K1*.

Since different radiological features were also made up of various histological subtypes that might contribute to their molecular heterogeneity, we further analyzed the radiological and molecular features among patients with only MIA and IAC. Of the 479 patients with MIA tumors, 53.0% (*n* = 254) had pure GGOs, 43.6% (*n* = 209) had mixed GGOs, and 2.9% (*n* = 14) had solid pulmonary nodules. Meanwhile, among the 2,523 patients with IAC, 3.0% (*n* = 75) had pure GGOs, 47.1% (*n* = 1,189) had mixed GGOs, and 48.8% (*n* = 1,232) had solid pulmonary nodules. Mutation rates were similar between MIA and IAC that appeared as pure GGOs and mixed GGOs and were very different from tumors that appeared as solid nodules (Fig. S13C, D). Further analysis on the distribution of MEA according to radiological features in MIA (*P* = 0.53; Fig. S13E) and IAC (*P* = 0.94; Fig. S13F) did not show statistical significance.

## Discussion

LUAD is a molecularly and histologically heterogeneous disease. A better understanding of the molecular mechanisms by which somatic mutations contribute to the unique histopathologic and radiologic features of LUAD is the key to identifying biomarkers and developing effective strategies for diagnosis and treatment to improve the prognosis of patients with LUAD. To the best of our knowledge, our retrospective study is the first to include the largest cohort of Chinese patients to investigate the histopathologic, molecular, and radiologic characteristics of surgically-resected LUAD in our population. The use of resected tumor samples ensures the accurate histological evaluation and classification of these LUAD subtypes, which provides a better representation of the prevalent LUAD subtypes among the Chinese population. Moreover, the inclusion of a large cohort enabled further investigations on distinct mutational features between preinvasive and invasive LUAD that may be potential molecular mechanisms that drive the malignant transformation of preinvasive lesions. Our main findings identified a group of gene mutations that were frequently mutated in AIS, including *MAP2K1* indels, *BRAF* non-V600E kinase mutations, and 20ins in both *EGFR* and *ERBB2*, which we referred to as MEA.

Based on our findings, *EGFR* and *TP53* mutations were detected from most tumors having an increasing trend concomitant with increased invasiveness, resulting in more predominant mutations in IAC (*EGFR*: 30.1% vs. 56.4% vs. 71.5%; *TP53*: 0.8% vs. 4.2% vs. 36.2%). Meanwhile, the mutations in genes, including *ERBB2*, *BRAF*, and *MAP2K1*, which were mutually exclusive from *EGFR* mutations, had the opposite trend and were found more predominantly in preinvasive lesions, and least detected in IAC (*ERBB2*: 23.2% vs. 15.7% vs. 5.2%; *BRAF*: 16.7% vs. 5.9% vs. 2.1%; *MAP2K1*: 10.6% vs. 7.1% vs. 0.9%). Consistent with our findings, earlier studies have also reported that *BRAF* mutations are associated with early LUAD carcinogenesis, *EGFR* mutations are equally detected in preinvasive and invasive lesions, whereas *TP53* mutations may be late events associated with subclonal diversity and malignant progression^10,12,13^. Consistently, our pathway analysis suggested the role of three key pathways in different stages of malignant progression. ERBB pathway activation was constantly important throughout lung carcinogenesis; MAPK pathway activation regulates the early events of tumor development, and p53 pathway deactivation cooperates with other oncogenic drivers to promote malignant progression.

MAP2K1 or MEK1 is one of the genes involved in the MAPK pathway and is a downstream effector of RAF kinase^25–27^. MAP2K1 is rarely mutated in lung cancers (~1–2%) and implicated as an oncogenic driver in a small subset of LUAD that might benefit from MEK1 inhibitor therapy^25–27^. Genetic alterations in *MAP2K1*, such as E102_I103del and other small in-frame deletions between L98 and K104, were demonstrated to induce cell proliferation, differentiation, and transformation through constitutive kinase activity that is independent of RAF kinase^26,27^. These mutations are sensitive to ATP-competitive MEK1 inhibition but not to allosteric MEK1 inhibitors^26,27^. Pan-cancer analyses have identified several solid tumor types that harbor various unique in-frame indels in *MAP2K1*, with E102_I103del (6/14) being the most common^27^. In contrast to RAF-independent *MAP2K1*-mutants being mostly indels, RAF-dependent *MAP2K1*-mutants were mostly missense mutations and were associated with concurrent activating mutations in RAS, RAF, or other receptor tyrosine kinase genes^26^. This is in line with our findings that *MAP2K1* E102_I103del in AIS was more likely to occur as single mutations and might function as an oncogenic driver during the neoplastic stage, while *MAP2K1* missense mutations in IAC had more concurrent activating mutations and were more likely to be passenger mutations. It should be noted that this is the first clinical report of *MAP2K1* E102_I103del as a hotspot mutation in AIS; therefore, we are unsure whether this observation is only specific to our population or it is due to the large AIS cohort included in our cohort (*n* = 246) compared to earlier studies.

In addition, the mutational features associated with *BRAF* were also distinct between preinvasive and invasive tumors. *BRAF* is also an important regulator of the MAPK pathway, with mutations identified in 1–5% of lung cancer^24,28^. *BRAF* V600E is RAS-independent and able to constitutively induce cell proliferation, while *BRAF* non-V600E mutations could induce cell proliferation at varying degrees depending on their dependence on RAS activation^28^. Recent evidences have also shown that certain *BRAF* non-V600E mutations are responsive to MEK inhibitors, such as trametinib, with or without BRAF inhibitor (dabrafenib)^29^. According to the emerging classification system for *BRAF* mutations, V600E mutations signaling as RAS-independent active monomers are classified as Class I, while kinase non-V600E mutations identified in our cohort were of Class II and III, which function as RAS-independent activated dimers or RAS-dependent kinase-impaired or inactivated heterodimers^24,30^. Consistently, our data showed *BRAF* non-V600E kinase-mutant IAC had more co-occurring mutations in oncogenic driver genes and TSGs compared to V600E-mutant IAC, suggesting the weaker oncogenic activity of *BRAF* non-V600E mutations than V600E. Interestingly, a subset of AIS and MIA with *BRAF* non-V600E kinase mutations in our cohort harbored concurrent *TSC1* LOF mutations, which were independent of activating mutations in *KRAS* or other oncogenes. Whether the co-occurrence between *TSC1* mutations and *BRAF* non-V600E kinase mutations contributes to tumor development is unknown and deserves to be explored in pre-clinical studies.

NSCLCs harboring *EGFR* driver mutations benefit from first-generation to third-generation EGFR tyrosine kinase inhibitors (TKIs); however, *EGFR* 20ins tumors show significantly lower sensitivity^31,32^. The recent FDA accelerated approval of EGFR TKI mobocertinib^32^ and EGFR-MET-targeted bispecific antibody amivantamab^31^ for the treatment of advanced-stage NSCLC harboring *EGFR* 20ins had altered the therapeutic algorithm for these patients. Moreover, poziotinib was granted a fast track designation as a potential treatment for patients with *ERBB2* 20ins-mutant NSCLC^33^. However, a question of whether these positive clinical outcomes could translate in the adjuvant or neoadjuvant setting of early-stage lung cancer remains to be answered. *EGFR* and *ERBB2* 20ins were respectively detected in 1.6% and 1.4% of lung cancers from the American Association for Cancer Research Project GENIE datasets^34^. In our large cohort of preinvasive LUAD, *EGFR* and *ERBB2* 20ins were identified in 8.1% and 20.3% of AIS, suggesting their potential oncogenic roles in preinvasive lesions. Furthermore, the detection rate of co-occurring *PIK3CA* mutations was significantly higher in *EGFR* 20ins-mutant IAC, indicating the diverse biomedical and pathological processes. Other reports have also observed that *EGFR* 20ins co-occur with other mutations, including *PIK3CA*^35,36^. In our cohort, *EGFR* and *ERBB2* 20ins were more frequent in younger patients. Moreover, *EGFR* p.A767_V769dup and p.S768_D770dup were the two most prevalent mutation subtypes of *EGFR* 20ins, whereas *ERBB2* p.A775_G776insYVMA was the most prevalent mutation subtype of *ERBB2* 20ins. These findings were consistent with previous reports in lung cancer patients younger than 65 years and Chinese patients with lung cancer^36–40^. Furthermore, despite the lower mutation-positive rates for *EGFR* and *ERBB2* 20ins in IAC, the similar distribution of the predominant *EGFR* and *ERBB2* 20ins subtypes in preinvasive lesions and invasive tumors suggests that *EGFR* and *ERBB2* 20ins are predominant in preinvasive lesions regardless of their mutation subtypes. Overall, our observations suggest that the group of MEA (i.e. *MAP2K1* indel, *BRAF* non-V600E kinase, and 20ins in *EGFR* and *ERBB2*) could potentially be involved in early LUAD tumorigenesis.

Genomic and proteomic studies consistently suggest a step-wise transformation of preinvasive AIS lesions into invasive tumors^15,41^. A report by Hu et al implicated the progressive accumulation of SNVs in the genomic evolution of AAH to AIS, MIA, and IAC using multi-region exome sequencing^15^. Moreover, their data suggest a clonal sweep model as a molecular mechanism underlying the progression of lung preneoplasia. Our data also supports this trend, wherein significantly higher mutation counts and CNVs were detected in IAC than AIS or MIA (Fig. 2B, C), with a subset of mutations detected in AIS, such as the MEA, would eventually be eliminated and replaced by other stronger oncogenic driver mutations, including *EGFR* activating mutations L858R and 19del.

In addition to histopathological and molecular heterogeneity, the radiological appearance of lung cancer is also heterogeneous. Numerous reports have demonstrated the direct correlation between *EGFR* mutation rate and the proportion of solid component admixed with GGO^9,42–45^. Consistent with these reports, mutations in genes involved in lung cancer development were detected in tumors that appeared as GGOs; however, the overall mutation rates were lower compared to tumors that had solid components. Our findings on the pattern of *EGFR* mutation rates in MIA and IAC that were higher in solid solitary nodules and lowest in pure GGOs are in line with previous findings on the detection of *EGFR* mutation in GGOs, which showed the direct correlation between *EGFR* mutation rate and the proportion of solid component admixed with GGO^9,42–45^.

Despite the inclusion of a large cohort of patients, our study is limited by being conducted in a single institution, which introduces patient selection bias. Our study only used a small targeted gene panel to investigate the genetic aspect of LUAD, which could miss other key mutations important in early carcinogenesis. It would also be interesting to conduct a multi-omics study to investigate the epigenomics, proteomics, and the distinct features of the tumor microenvironment in various LUAD subtypes. In addition, the retrospective nature of our study limits our data analysis due to the lack of data on therapeutic management and survival outcomes. It would be clinically relevant to explore the survival outcome differences among certain molecular subsets of LUAD.

In conclusion, our study advances our current understanding of the molecular, radiological, and histopathological profiles of resectable LUAD. The unique somatic mutation landscape of various histological subtypes of LUAD provides insights into lung cancer pathogenesis as well as the need for individualized clinical management of patients. Furthermore, our data demonstrate the distinct mutational features between preinvasive lesions and invasive tumors with MEA, suggesting the potential involvement of MEA in the early stages of tumorigenesis. Further pre-clinical studies are required to establish the role of these genes in the malignant transformation of preinvasive lesions into invasive tumors.

## Supplementary information

Supplementary materials

## Data Availability

All authors confirm adherence to the policy. The data that support the findings of this study will be made available at reasonable request. Correspondence and requests for data and materials should be addressed to YH.
