# Corrigendum to “Distinct mutational features across preinvasive and invasive subtypes identified through comprehensive profiling of surgically resected lung adenocarcinoma” [*Modern Pathology*. 2022;35(9):1181-1192]

**DOI:** 10.1016/j.modpat.2023.100407

**Published:** 2024-01-05

**Authors:** Chan Xiang, Chunyu Ji, Yiran Cai, Haohua Teng, Yulu Wang, Ruiying Zhao, Zhanxian Shang, Lianying Guo, Shengnan Chen, Analyn Lizaso, Jing Lin, Haozhe Wang, Bing Li, Zhou Zhang, Jikai Zhao, Jinzhi Wei, Jiaxin Liu, Lei Zhu, Wentao Fang, Yuchen Han

**Affiliations:** a Department of Pathology, Shanghai Chest Hospital, Shanghai Jiao Tong University, 200030, Shanghai, China; b Department of Thoracic Surgery, Shanghai Chest Hospital, Shanghai Jiao Tong University, 200030, Shanghai, China; c Burning Rock Biotech, 510300, Guangzhou, China

The authors of the article entitled, “Distinct mutational features across preinvasive and invasive subtypes identified through comprehensive profiling of surgically resected lung adenocarcinoma” (*Modern Pathology* 2022;35(9):1181-1192; DOI: https://doi.org/10.1038/s41379-022-01076-w) have discovered an error in the published Acknowledgements statement related to the funding number of the National Natural Science Foundation of China by CX. The correct number should be as follows: 82003154. A corrected statement in full is included below.

Corrected acknowledgements in full:

The authors thank all the patients who participated in this study and their families. We also thank the investigators, study coordinators, operation staff, and the whole project team who worked on this study. This work was supported by grants from the Shanghai Municipal Health Commission Intelligent Medical Research Project (Grant no. 2018ZHYL0213 to YH); Clinical Research Project (Grant No. 20194Y0053 to CX); National Natural Science Foundation of China (Grant No. 82003154 to CX); and Shanghai Jiao Tong University Medical Engineering Cross Fund (Grant no. YG2019QNA50 to CX). The funders had no role in the study design, participant recruitment, data collection, data analysis, data interpretation, manuscript preparation, and the decision to submit the paper for publication.

