# Supplementary material for: Distinct mutational features across preinvasive and invasive subtypes identified through comprehensive profiling of surgically resected lung adenocarcinoma
Source: Mod Pathol. 2022 May 31;35(9):1181–92. doi: 10.1038/s41379-022-01076-w (PMC9424111; doi:10.1038/s41379-022-01076-w)
Supplement: Supplementary file 1 — Supplementary materials [file 41379_2022_1076_MOESM1_ESM.pdf]

Supplementary files for

Distinct mutational features across pre-invasive and invasive subtypes identified through comprehensive profiling of surgically resected lung adenocarcinoma

Chan Xiang, Chunyu Ji, Yiran Cai, Haohua Teng, Yulu Wang, Ruiying Zhao, Zhanxian Shang, Lianying Guo, Shengnan Chen, Jing Lin, Analyn Lizaso, Haozhe Wang, Bing Li, Zhou Zhang, Jikai Zhao, Jinzhi Wei, Jiaxin Liu, Lei Zhu, Wentao Fang*, Yuchen Han*

*** Corresponding authors:**

Yuchen Han, MD, PhD; Department of Pathology, Shanghai Chest Hospital, Shanghai Jiao Tong University; 241 West Huaihai Road, Shanghai 200030, China. Tel: 86-21-2220-0000. Fax: 86-21-6280-1109. Email address: ychan@cmu.edu.cn

Wentao Fang, MD; Department of Thoracic Surgery, Shanghai Chest Hospital, Shanghai Jiao Tong University; 241 West Huaihai Road, Shanghai 200030, China. Tel: 86-21-2220-0000. Fax: 86-21-6280-1109. Email address: vwtfang@hotmail.com

Table of contents	Page number/File
**Supplementary Tables**	
Table S1. List of genes included in the 68-gene Lung Core panel.	Page 3
Table S2. Summary of the clinical, pathological, radiological, and molecular characteristics of the cohort according to histological subtypes.	Page 4-5
Table S3. Mutation detection rates of various genes in adenocarcinoma in situ (AIS) and invasive adenocarcinoma (IAC).	Page 6
Table S4. List of *MAP2K1* mutations detected in our cohort.	Page 7-8
Table S5. List of *BRAF* mutations detected in our cohort.	Page 9-10
**Supplementary figures**	
Figure S1. Difference in smoking history and tumor size distribution across histological subtypes.	Page 11
Figure S2. Molecular landscape of stage 0-IIIA LUAD.	Page 12
Figure S3. Distinct mutation types between preinvasive to minimally invasive adenocarcinomas, and invasive LUAD.	Page 13
Figure S4. Relationship analysis between genes and specific mutations across all tumors according to invasion levels.	Page 14
Figure S5. Signaling pathways involved in the acquisition of LUAD invasiveness.	Page 15
Figure S6. Mutational features of *MAP2K1* & *BRAF* in minimally invasive adenocarcinoma (MIA) and molecular features according to invasion levels.	Page 16
Figure S7. *MAP2K1* mutations were predominantly oncogenic/likely oncogenic.	Page 17
Figure S8. Distribution of *BRAF* mutations categorized according to kinase activity.	Page 18
Figure S9. Concurrent mutation landscape in *EGFR*-mutant and *ERBB2*-mutant lung lesions.	Page 19
Figure S10. Distribution of age, mutation count, tumor size, and smoking history in AIS, MIA, and IAC according to mutations enriched in AIS (MEA) status.	Page 20
Figure S11. Molecular features of IAC subtypes.	Page 21
Figure S12. Molecular features according to radiological characteristics.	Page 22
Figure S13. Pulmonary nodules characterized by either pure or mixed ground-glass opacities (GGO) have a similar mutation profile.	Page 23

Table S1. List of genes included in the 68-gene Lung Core panel.

AKT1	*CD74*	*ESR1*	*JAK1*	*NRG1*	*SMAD4*
ALK	*CDK4*	*FGF3*	*JAK2*	*NTRK1*	*SMO*
APC	*CDK6*	*FGF4*	*KDR*	*NTRK2*	*STK11*
AR	*CDKN2A*	*FGF19*	*KIT*	*NTRK3*	*TOP2A*
ARAF	*CTNNB1*	*FGFR1*	*KRAS*	*PDGFRA*	*TP53*
ATM	*CYP2D6*	*FGFR2*	*MAP2K1*	*PIK3CA*	*TSC1*
AXL	*DDR2*	*FGFR3*	*MET*	*PTCH1*	*TSC2*
BCL2L11	*DPYD*	*FLT3*	*MTOR*	*PTEN*	*UGT1A1*
BRAF	*EGFR*	*HRAS*	*MYC*	*RAF1*	
BRCA1	*ERBB2*	*IDH1*	*NF1*	*RB1*	
BRCA2	*ERBB3*	*IDH2*	*NOTCH1*	*RET*	
CCND1	*ERBB4*	*IGF1R*	*NRAS*	*ROS1*	

Table S2. Summary of the clinical, pathological, radiological, and molecular characteristics of the cohort according to histological subtypes

Features	Preinvasive lesions (n,%)			Invasive adenocarcinoma (n=2,523) (n,%)								
	AAH (n=6)	AIS (n=246)	MIA (n=479)	Lepidic (n=301)	Acinar (n=1,367)	Papillary (n=412)	Micropapillary (n=93)	Solid (n=243)	Invasive Mucinous (n=85)	Enteric (n=18)	Fetal (n=3)	Colloid (n=1)
**Pathological stage**												
NA (AAH)	6(100%)	0(0%)	0(0%)	0(0%)	0(0%)	0(0%)	0(0%)	0(0%)	0(0%)	0(0%)	0(0%)	0(0%)
stage 0	0(0%)	246(100%)	0(0%)	0(0%)	0(0%)	0(0%)	0(0%)	0(0%)	0(0%)	0(0%)	0(0%)	0(0%)
stage IA-B	0(0%)	0(0%)	479(100%)	300(99.7%)	1125(82.3%)	319(77.4%)	42(45.2%)	107(44.0%)	54(63.5%)	8(44.4%)	2(66.7%)	0(0%)
stage IIA-B	0(0%)	0(0%)	0(0%)	1(0.3%)	101(7.4%)	48(11.7%)	12(12.9%)	46(18.9%)	17(20%)	5(27.8%)	1(33.3%)	0(0%)
stage IIIA	0(0%)	0(0%)	0(0%)	0(0%)	141(10.3%)	45(10.9%)	39(41.9%)	90(37.0%)	14(16.5%)	5(27.8%)	0(0%)	1(100%)
**Radiological features of nodule**												
Pure GGO	5(83.3%)	210(85.4%)	254(53%)	30(10.0%)	38(2.8%)	7(1.7%)	0(0%)	0(0%)	0(0%)	0(0%)	0(0%)	0(0%)
Mixed GGO	1(16.7%)	33(13.4%)	209(43.6%)	262(87.0%)	718(52.5%)	160(38.8%)	14(15.1%)	11(4.5%)	23(27.1%)	1(5.6%)	0(0%)	0(0%)
Solid nodule	0(0%)	3(1.2%)	14(2.9%)	9(3.0%)	593(43.4%)	240(58.3%)	79(84.9%)	229(94.2%)	61(71.8%)	17(94.4%)	3(100%)	1(100%)
**Overall mutation rate**	4(66.7%)	240(97.6%)	470(98.1%)	298(99.0%)	1,363(99.7%)	410(99.5%)	93(100%)	239(98.4%)	85(100%)	18(100%)	3(100%)	1(100%)
**CNV varscore (median, [range minimum, maximum]**	0	1[1,2]	1[1,2]	1[1,3]	1[1,6]	1[1,6]	1[1,3]	1[1,7]	1[1,4]	2[1,5]	1.5[1,2]	0
**Mutation rate**												
ALK fusion	0(0%)	2(0.8%)	5(1%)	3(1.0%)	37(2.7%)	9(2.2%)	7(7.5%)	26(10.7%)	10(11.8%)	0(0%)	0(0%)	0(0%)
EGFR exon 19 deletions	0(0%)	19(7.7%)	93(19.4%)	77(25.6%)	411(30.1%)	155(37.6%)	25(26.9%)	34(14.0%)	3(3.5%)	3(16.7%)	0(0%)	0(0%)
EGFR exon 19 insertions	0(0%)	1(0.4%)	1(0.2%)	1(0.3%)	1(0.1%)	1(0.2%)	1(1.1%)	0(0%)	0(0%)	0(0%)	0(0%)	0(0%)
EGFR exon 20 insertions	1(16.7%)	20(8.1%)	20(4.2%)	10(3.3%)	48(3.5%)	15(3.6%)	5(5.4%)	2(0.8%)	0(0%)	0(0%)	0(0%)	0(0%)
EGFR G719X	0(0%)	5(2.0%)	10(2.1%)	0(0%)	45(3.3%)	11(2.7%)	4(4.3%)	3(1.2%)	0(0%)	0(0%)	0(0%)	0(0%)
EGFR L858R	0(0%)	24(9.8%)	135(28.2%)	149(49.5%)	538(39.4%)	121(29.4%)	26(28.0%)	27(11.1%)	2(2.4%)	4(22.2%)	0(0%)	0(0%)
EGFR L861Q	0(0%)	1(0.4%)	6(1.3%)	2(0.7%)	30(2.2%)	6(1.5%)	0(0%)	2(0.8%)	0(0%)	0(0%)	0(0%)	0(0%)
EGFR S768I	0(0%)	3(1.2%)	4(0.8%)	1(0.3%)	12(0.9%)	3(0.7%)	2(2.2%)	0(0%)	0(0%)	0(0%)	0(0%)	0(0%)
EGFR T790M	0(0%)	0(0%)	3(0.6%)	2(0.7%)	15(1.1%)	4(1%)	0(0%)	2(0.8%)	0(0%)	0(0%)	0(0%)	0(0%)

Table S2 continued

Features	Preinvasive lesions (n,%)			Invasive adenocarcinoma (n=2,523) (n,%)								
	AAH (n=6)	AIS (n=246)	MIA (n=479)	Lepidic (n=301)	Acinar (n=1,367)	Papillary (n=412)	Micropapillary (n=93)	Solid (n=243)	Invasive Mucinous (n=85)	Enteric (n=18)	Fetal (n=3)	Colloid (n=1)
**Mutation rate**												
ERBB2 exon 20 insertion	1(16.7%)	50(20.3%)	67(14.0%)	9(3.0%)	35(2.6%)	17(4.1%)	2(2.2%)	8(3.3%)	2(2.4%)	3(16.7%)	0(0%)	0(0%)
ERBB2 amplification	0(0%)	0(0%)	0(0%)	1(0.3%)	7(0.5%)	4(1.0%)	1(1.1%)	2(0.8%)	1(1.2%)	1(5.6%)	0(0%)	0(0%)
ERBB2 S310	0(0%)	0(0%)	2(0.4%)	1(0.3%)	5(0.4%)	0(0%)	1(1.1%)	1(0.4%)	0(0%)	0(0%)	0(0%)	0(0%)
KRAS G12	0(0%)	13(5.3%)	13(2.7%)	7(2.3%)	63(4.6%)	25(6.1%)	10(10.8%)	34(14.0%)	58(68.2%)	3(16.7%)	1(33.3%)	0(0%)
KRAS G13	0(0%)	1(0.4%)	1(0.2%)	0(0%)	4(0.3%)	0(0%)	1(1.1%)	5(2.1%)	2(2.4%)	0(0%)	0(0%)	0(0%)
KRAS Q61	0(0%)	1(0.4%)	2(0.4%)	1(0.3%)	1(0.1%)	4(1.0%)	1(1.1%)	4(1.6%)	2(2.4%)	0(0%)	0(0%)	0(0%)
MET exon 14 splicing	0(0%)	2(0.8%)	11(2.3%)	10(3.3%)	16(1.2%)	5(1.2%)	0(0%)	5(2.1%)	0(0%)	0(0%)	0(0%)	0(0%)
MET amplification	0(0%)	0(0%)	0(0%)	0(0%)	9(0.7%)	9(2.2%)	1(1.1%)	8(3.3%)	1(1.2%)	0(0%)	0(0%)	0(0%)
RET fusion	0(0%)	1(0.4%)	10(2.1%)	8(2.7%)	30(2.2%)	4(1.0%)	1(1.1%)	9(3.7%)	0(0%)	0(0%)	0(0%)	0(0%)
ROS1 fusion	0(0%)	2(0.8%)	2(0.4%)	1(0.3%)	10(0.7%)	10(2.4%)	3(3.2%)	11(4.5%)	0(0%)	0(0%)	0(0%)	0(0%)
BRAF V600E	0(0%)	0(0%)	1(0.2%)	1(0.3%)	8(0.6%)	1(0.2%)	2(2.2%)	2(0.8%)	0(0%)	0(0%)	0(0%)	0(0%)
BRAF non-V600E	1(16.7%)	41(16.7)	27(5.6%)	2(0.7%)	19(1.4%)	7(1.7%)	0(0%)	9(3.7%)	1(1.2%)	1(5.6%)	0(0%)	0(0%)
MAP2K1 E102_I103del	0(0%)	17(6.9%)	26(5.4%)	1(0.3%)	1(0.1%)	0(0%)	0(0%)	0(0%)	0(0%)	0(0%)	0(0%)	0(0%)
MAP2K1 other indel	0(0%)	7(2.8%)	6(1.3%)	0(0%)	1(0.1%)	0(0%)	0(0%)	1(0.4%)	0(0%)	0(0%)	0(0%)	0(0%)
MAP2K1 missense mutations	0(0%)	2(0.8%)	2(0.4%)	0(0%)	9(0.7%)	3(0.7%)	1(1.1%)	3(1.2%)	1(1.2%)	1(5.6%)	0(0%)	0(0%)

Abbreviations: AAH, atypical adenomatous hyperplasia; AIS, adenocarcinoma in situ; CNV, copy number variation; GGO, ground glass nodules; MIA, minimally invasive adenocarcinoma; del, deletion; indel, insertion-deletion variation

Table S3. Mutation detection rates of various genes in adenocarcinoma in situ (AIS) and invasive adenocarcinoma (IAC). Related to Figure 2F.

**Genes**	**Mutation detection rates (%)**		***p*-values**	**False discovery rate-corrected *p*-values**
	**AIS**	**IAC**		
EGFR Other	21.95	68.9	5.9596E-47	2.0263E-45
ERBB2 exon 20 insertion	20.33	3.0	2.9218E-22	2.4835E-21
BRAF non-V600E	16.67	1.3	5.7147E-25	6.4766E-24
MAP2K1	10.57	0.9	1.206E-15	8.2007E-15
EGFR exon 20 insertion	8.13	3.2	0.00042451	0.00144335
KRAS	7.72	9.7	0.36300456	0.42798725
TSC1	4.88	1.9	0.00941432	0.02133913
ERBB2 Other	3.25	2.3	0.38059658	0.43134279
ALK	2.85	5.9	0.0571517	0.08832535
ATM	2.85	4.4	0.32115488	0.40441726
NF1	2.85	3.6	0.71671876	0.73843751
TSC2	2.85	2.8	1	1
CTNNB1	2.03	3.5	0.26930357	0.3521662
MTOR	2.03	2.9	0.68239508	0.72504477
PTCH1	2.03	1.5	0.42712822	0.46846321
TP53	0.81	36.1	6.1896E-42	1.0522E-40
CDK4	0.00	5.5	3.7721E-06	2.1375E-05
APC	1.63	4.9	0.01585575	0.03171151
PIK3CA	0.00	4.7	2.0343E-05	9.8807E-05
ROS1	1.22	4.5	0.01147264	0.02437936
RB1	0.00	4.2	7.4694E-05	0.00031745
MET	1.22	4.1	0.02249157	0.04024807
BRCA2	0.81	3.9	0.00713105	0.01865043
MYC	1.22	3.8	0.03088528	0.05250497
STK11	0.00	3.8	0.00017544	0.00066278
RET	0.81	3.4	0.0213367	0.04024807
SMAD4	0.00	2.9	0.00246955	0.00763316
CDKN2A	1.22	2.7	0.20515223	0.29063232
NOTCH1	0.41	2.5	0.04104835	0.06645923
PTEN	0.00	2.5	0.00552239	0.01564677
NTRK3	1.22	2.3	0.36504795	0.42798725
DDR2	0.00	2.3	0.008171	0.01984387
ERBB3	0.81	2.1	0.22991302	0.31268171
ERBB4	0.41	2.1	0.08542455	0.12627977

Note: Red font denotes statistical significance in both *p*-value and false discovery rate (FDR)-corrected *p*-values.

Table S4. List of *MAP2K1* mutations detected in our cohort.

Gene	Gene variant	Mutation type	Oncogenicity subgrouping	Total number of patients detected with particular variant	AAH	AIS	MIA	IAC
MAP2K1	E102_I103del	Indel	O/LO	45	0	17	26	2
MAP2K1	F53_Q58delinsL	Indel	O/LO	5	0	2	1	2
MAP2K1	Q58_E62del	Indel	O/LO	3	0	1	2	0
MAP2K1	L375I	Missense	VUS	2	0	0	0	2
MAP2K1	Q56P	Missense	O/LO	2	0	1	0	1
MAP2K1	D208N	Missense	VUS	1	0	0	0	1
MAP2K1	D67N	Missense	O/LO	1	0	0	0	1
MAP2K1	E102_K104delinsQ	Indel	VUS	1	0	1	0	0
MAP2K1	E144Q	Missense	VUS	1	0	0	0	1
MAP2K1	E163Q	Missense	VUS	1	0	0	0	1
MAP2K1	E41_F53del	Indel	O/LO	1	0	0	1	0
MAP2K1	E51_Q58del	Indel	O/LO	1	0	1	0	0
MAP2K1	G128D	Missense	O/LO	1	0	0	1	0
MAP2K1	H100_I103delinsRY	Indel	VUS	1	0	1	0	0
MAP2K1	I103N	Missense	O/LO	1	0	0	0	1
MAP2K1	I112L	Missense	VUS	1	0	0	0	1
MAP2K1	K57N	Missense	O/LO	1	0	0	1	0
MAP2K1	K57T	Missense	O/LO	1	0	0	0	1
MAP2K1	K5N	Missense	VUS	1	0	0	0	1
MAP2K1	L197V	Missense	VUS	1	0	0	0	1
MAP2K1	P105_A106del	Indel	VUS	1	0	0	1	0
MAP2K1	P105_I107delinsV	Indel	VUS	1	0	0	1	0
MAP2K1	P124S	Missense	O/LO	1	0	0	0	1
MAP2K1	Q110H	Missense	VUS	1	0	0	0	1
MAP2K1	Q354H	Missense	VUS	1	0	0	0	1
MAP2K1	Q56_V60del	Indel	O/LO	1	0	1	0	0
MAP2K1	S327I	Missense	VUS	1	0	0	0	1
MAP2K1	T55A	Missense	VUS	1	0	0	0	1

MAP2K1	Y130C	Missense	O/LO	1	0	0	0	1
MAP2K1	Y130H	Missense	O/LO	1	0	1	0	0

Abbreviations: O/LO, oncogenic/likely oncogenic. VUS, variants of unknown significance.

Table S5. List of *BRAF* mutations detected in our cohort.

Gene	Gene variant	Mutation type	BRAF_class subgrouping	BRAF_position subgrouping	Total number of patients detected with particular variant	AAH	AIS	MIA	IAC
BRAF	p.G469A	Missense	II	BRAF_Kinase	16	1	5	4	6
BRAF	p.V600E	Missense	I	BRAF_V600E	15	0	0	1	14
BRAF	p.K601E	Missense	II	BRAF_Kinase	11	0	4	5	2
BRAF	p.D594G	Missense	III	BRAF_Kinase	10	0	5	4	1
BRAF	p.N581S	Missense	III	BRAF_Kinase	9	0	4	3	2
BRAF	p.D594N	Missense	III	BRAF_Kinase	5	0	3	0	2
BRAF	p.L597R	Missense	II	BRAF_Kinase	4	0	4	0	0
BRAF	p.T599dup	Indel	II	BRAF_Kinase	4	0	4	0	0
BRAF	p.L597Q	Missense	II	BRAF_Kinase	3	0	2	0	1
BRAF	p.G466A	Missense	III	BRAF_Kinase	2	0	0	1	1
BRAF	p.G466V	Missense	III	BRAF_Kinase	2	0	0	1	1
BRAF	p.G469V	Missense	II	BRAF_Kinase	2	0	2	0	0
BRAF	p.A17_N20del	Indel	unknown	BRAF_Non_Kinase	1	0	0	0	1
BRAF	p.A598_T599insR	Indel	unknown	BRAF_Kinase	1	0	0	1	0
BRAF	p.D40E	Missense	unknown	BRAF_Non_Kinase	1	0	0	0	1
BRAF	p.D555G	Missense	unknown	BRAF_Kinase	1	0	0	1	0
BRAF	p.D594E	Missense	III	BRAF_Kinase	1	0	1	0	0
BRAF	p.E451K	Missense	unknown	BRAF_Non_Kinase	1	0	0	1	0
BRAF	p.E501*	Stop_gained	unknown	BRAF_Kinase	1	0	0	0	1
BRAF	p.E501V	Missense	unknown	BRAF_Kinase	1	0	0	0	1
BRAF	p.E695K	Missense	unknown	BRAF_Kinase	1	0	0	0	1
BRAF	p.E83*	Stop_gained	unknown	BRAF_Non_Kinase	1	0	0	0	1
BRAF	p.G209A	Missense	unknown	BRAF_Non_Kinase	1	0	0	0	1
BRAF	p.G32_A33dup	Indel	unknown	BRAF_Non_Kinase	1	0	0	0	1
BRAF	p.G464A	Missense	II	BRAF_Kinase	1	0	0	0	1
BRAF	p.G469R	Missense	II	BRAF_Kinase	1	0	1	0	0
BRAF	p.G469S	Missense	unknown	BRAF_Kinase	1	0	1	0	0
BRAF	p.G596R	Missense	III	BRAF_Kinase	1	0	0	0	1

BRAF	p.K483E	Missense	III	BRAF_Kinase	1	0	0	1	0
BRAF	p.K55Q	Missense	unknown	BRAF_Non_Kinase	1	0	0	0	1
BRAF	p.K601N	Missense	II	BRAF_Kinase	1	0	0	0	1
BRAF	p.L222fs	Frameshift	unknown	BRAF_Non_Kinase	1	0	0	0	1
BRAF	p.L485W	Missense	II	BRAF_Kinase	1	0	0	1	0
BRAF	p.M689I	Missense	unknown	BRAF_Kinase	1	0	0	0	1
BRAF	p.N486_P492delinsTL	Indel	unknown	BRAF_Kinase	1	0	0	1	0
BRAF	p.N581I	Missense	III	BRAF_Kinase	1	0	0	1	0
BRAF	p.N581T	Missense	III	BRAF_Kinase	1	0	1	0	0
BRAF	p.P345H	Missense	unknown	BRAF_Non_Kinase	1	0	0	0	1
BRAF	p.R424L	Missense	unknown	BRAF_Non_Kinase	1	0	0	1	0
BRAF	p.R506_K507insVLR	Indel	unknown	BRAF_Kinase	1	0	1	0	0
BRAF	p.R719H	Missense	unknown	BRAF_Non_Kinase	1	0	0	0	1
BRAF	p.S364L	Missense	unknown	BRAF_Non_Kinase	1	0	0	0	1
BRAF	p.S365A	Missense	unknown	BRAF_Non_Kinase	1	0	0	0	1
BRAF	p.T488_P492del	Indel	II	BRAF_Kinase	1	0	1	0	0
BRAF	p.T488_Q493delinsE	Indel	unknown	BRAF_Kinase	1	0	1	0	0
BRAF	p.T599_V600insR	Indel	unknown	BRAF_Kinase	1	0	0	0	1
BRAF	p.V600_K601delinsE	Indel	II	BRAF_Kinase	1	0	1	0	0
BRAF	p.W216*	Stop_gained	unknown	BRAF_Non_Kinase	1	0	0	0	1
BRAF	p.W619C	Missense	unknown	BRAF_Kinase	1	0	0	0	1
BRAF	p.Y78F	Missense	unknown	BRAF_Non_Kinase	1	0	0	0	1

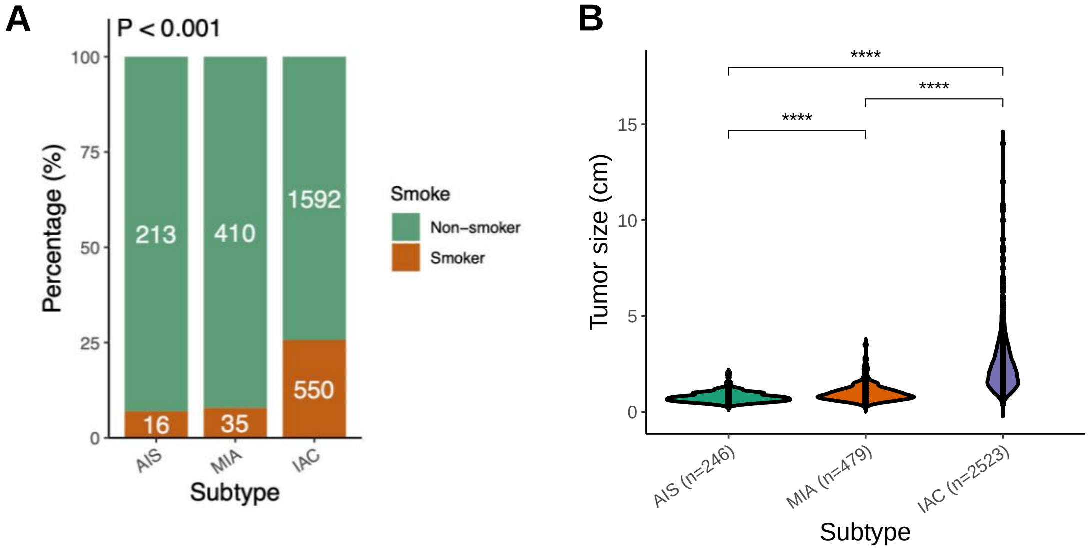

Figure S1. **Difference in smoking history and tumor size distribution across histological subtypes**. **A**. Component bar plot illustrating the distribution of smokers and non-smokers with AIS, MIA, and IAC. **B**. Violin plot illustrating the significantly different tumor sizes in adenocarcinoma in situ (AIS), minimally invasive adenocarcinoma (MIA), and invasive adenocarcinoma (IAC). Asterisks denote statistical significance, wherein **** represents P<0.0001.

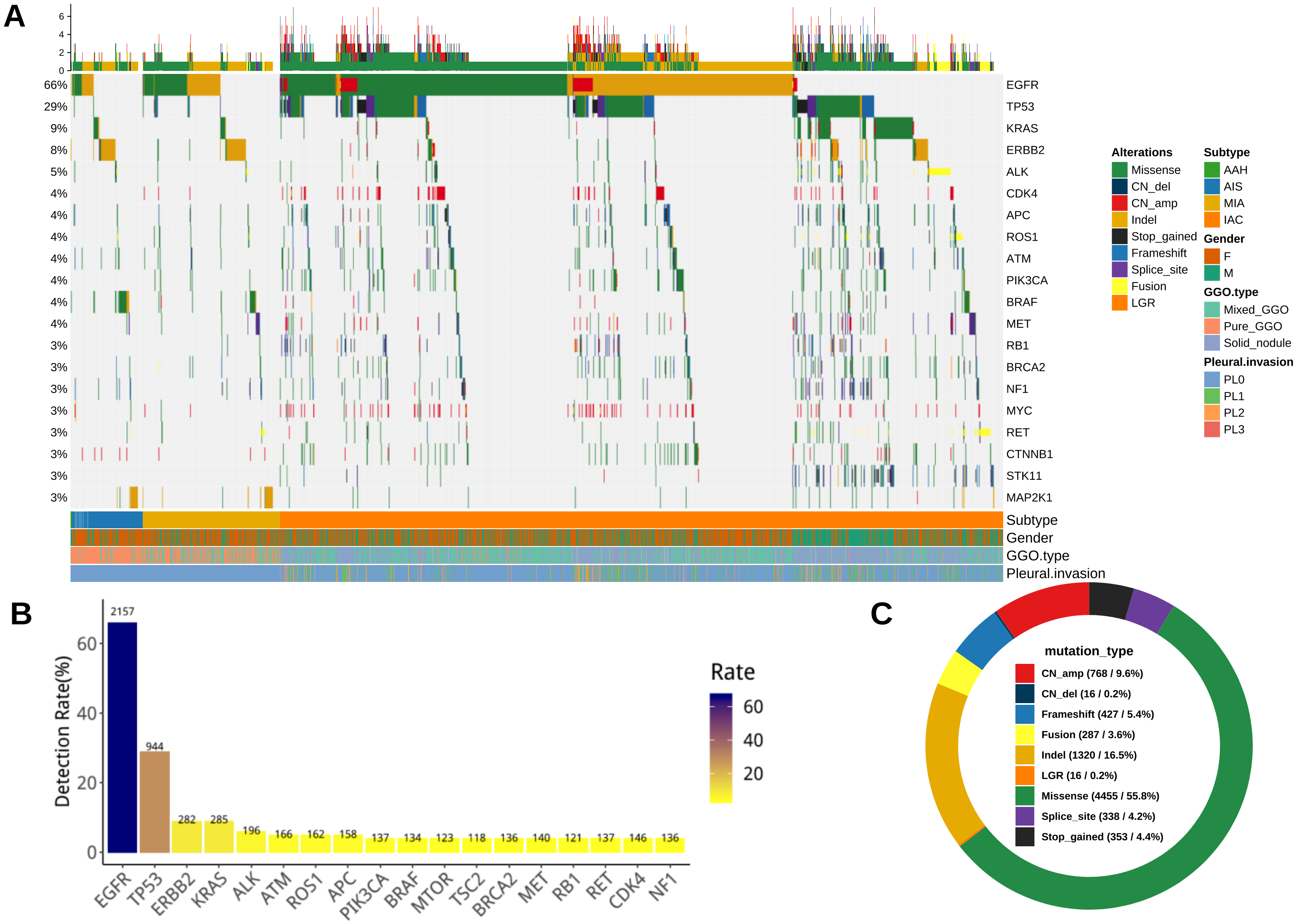

Figure S2. **Molecular landscape of stage 0-IIIA lung adenocarcinoma**. **A**. Oncoprint illustrating the mutation profile of our cohort. The histological subtypes, radiological phenotypes (i.e. ground glass opacity (GGO) type), visceral pleural (PL) invasion status, and gender [i.e. female (F) or male (M)] of each patient are indicated by various colors at the bottom of the oncoprint. Each column represents a patient and each row represents a gene. Values on the left represent the percentage of patients with mutations in a specific gene indicated on the right. Plot on top represents the overall number of mutations a patient harbored. Different colors denote the mutation types, including missense mutation, copy number deletion (CN_del), copy number amplification (CN_amp), insertion-deletion (indel), stop gained, frameshift, splice site variants, fusion, and large genomic rearrangements (LGR). **B**. Histogram summarizing the distribution of mutation rate of various genes detected from our cohort. **C**. Donut chart illustrating the distribution of mutation types detected from our cohort.

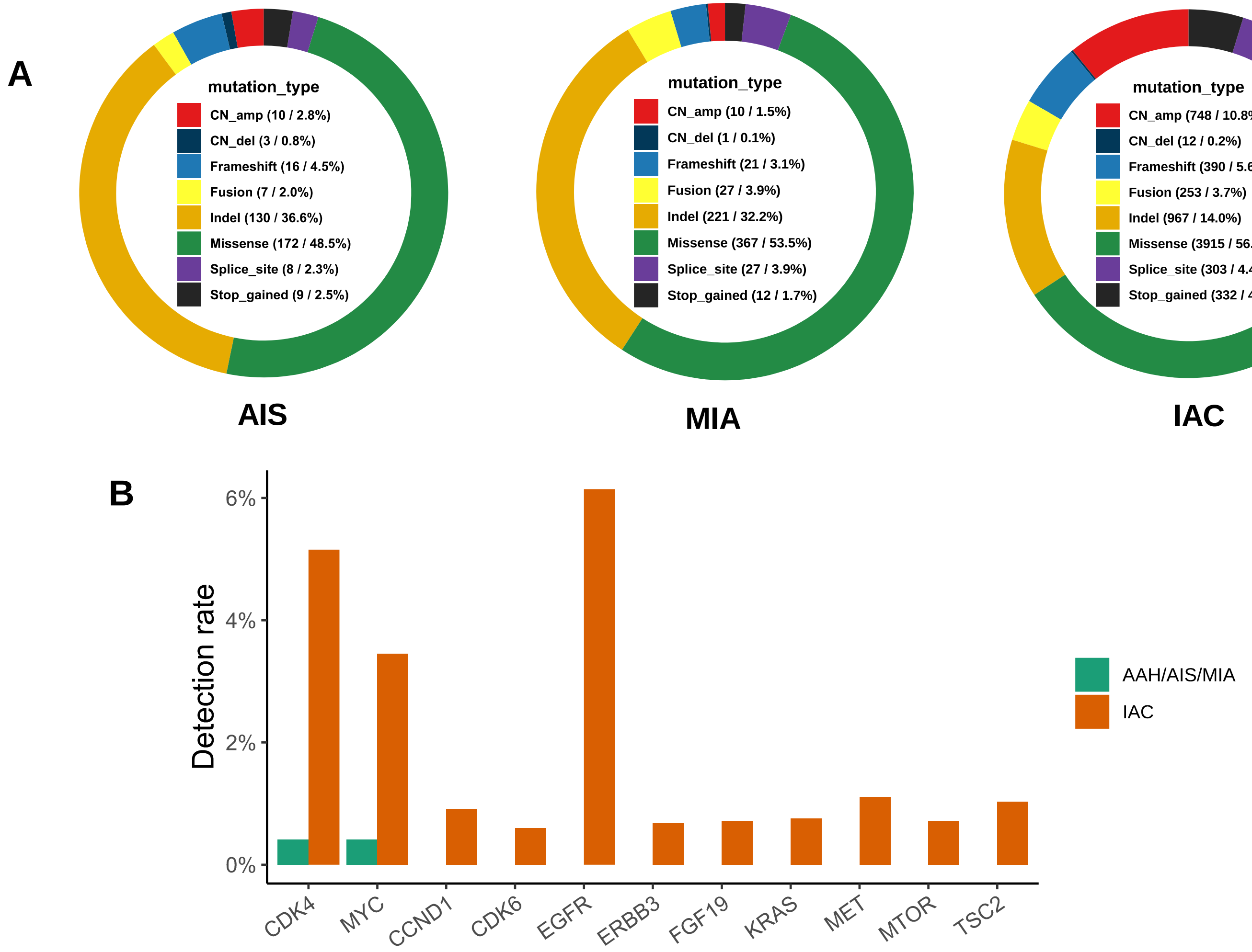

Figure S3. Distinct mutation types between preinvasive to minimally invasive adenocarcinomas, and invasive LUAD. A. Donut charts illustrating the distribution of mutation types detected from the three main histological subtypes, adenocarcinoma in situ (AIS), minimally invasive adenocarcinoma (MIA), and invasive adenocarcinoma (IAC). Different colors denote the mutation types, including missense mutation, copy number deletion (CN_del), copy number amplification (CN_amp), insertion-deletion (indel), stop gained, frameshift, splice site variants, fusion, and large genomic rearrangements (LGR). **B**. Histogram comparing the distribution of copy number variations (CNV) in preinvasive/minimally invasive lesions (AAH, AIS, MIA), and invasive LUAD (IAC).

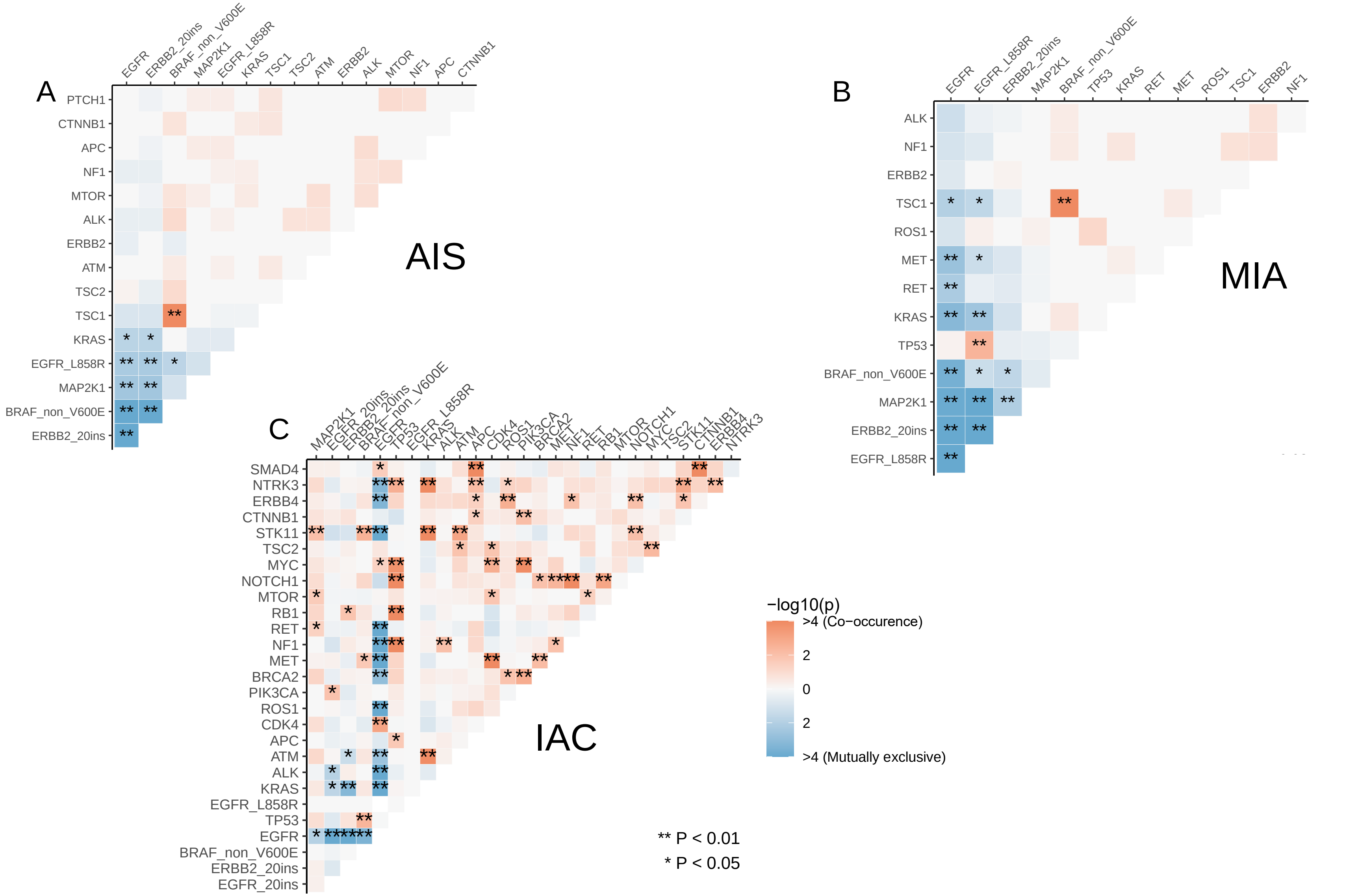

Figure S4. Relationship analysis between genes and specific mutations across all tumors according to invasion levels. A-C. Heat maps showing the mutually exclusive and co-occurring relationship among gene mutations in adenocarcinoma in situ (AIS) (**A**), minimally invasive adenocarcinoma (MIA) (**B**), and invasive adenocarcinoma (IAC) (**C**). Abbreviation: 20ins, exon 20 insertions

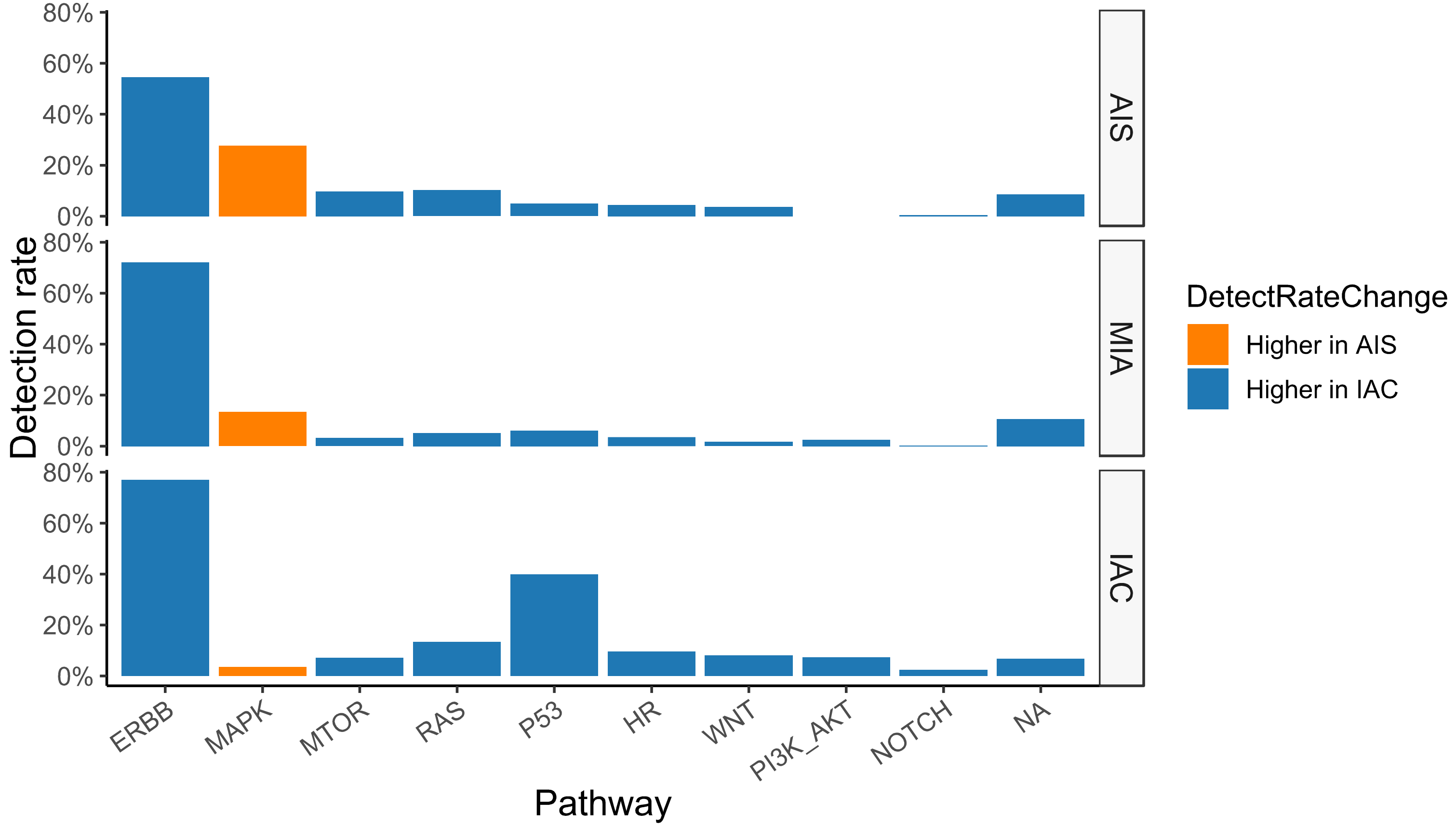

Figure S5. **Signaling pathways involved in the acquisition of LUAD invasiveness.** Faceted bar plots illustrating the differential rates of mutation detection in various signaling pathways of the three main subtypes adenocarcinoma in situ (AIS), minimally invasive adenocarcinoma (MIA), and invasive adenocarcinoma (IAC). Blue bars indicate a pattern of higher mutation rates in IAC and lower in AIS. Orange bars indicate a pattern of higher mutation rates in AIS and lower in IAC.

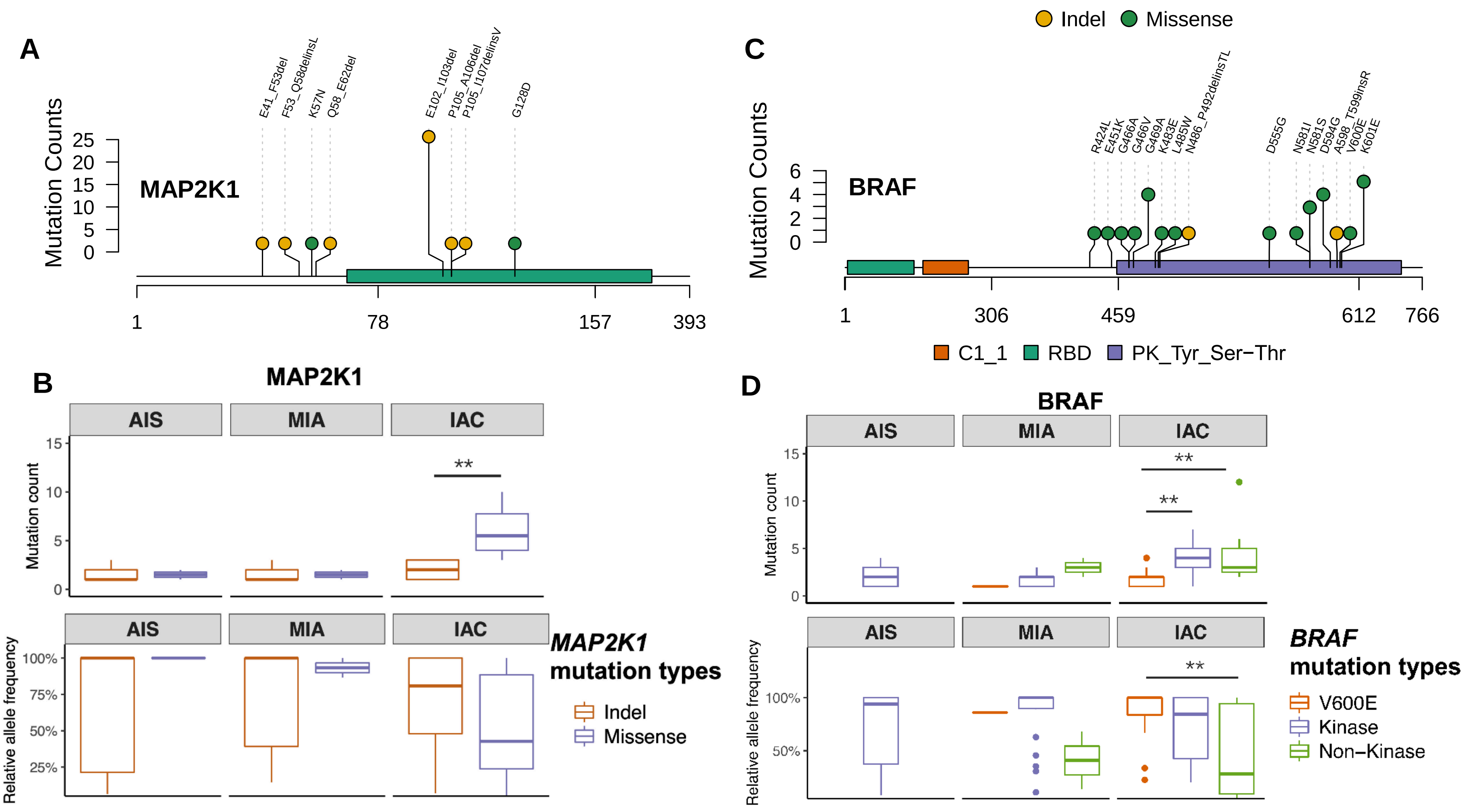

Figure S6. Mutational features of *MAP2K1* & *BRAF* in minimally invasive adenocarcinoma (MIA) and molecular features according to invasion levels. A, C. Lollipop plots summarizing the mutation sites and mutation types in *MAP2K1* (**A**) and *BRAF* (**C**) detected in MIA tumors. Green dots represent missense mutations. Yellow dots represent insertion-deletion (Indel) mutations. Each dot denotes a mutation in the specific site. The height of the lollipop indicate the mutation count. **B, D**. Box plots illustrating the distribution of mutation count (top) and relative allele frequency (bottom) in tumors harboring various mutation subtypes of *MAP2K1* (**B**) and *BRAF* (**D**). Mutation count was the total number of mutations per sample. Relative allele frequency for each mutation per sample was calculated as the ratio between allele frequency of the mutation and the maximum somatic allele frequency (MSAF) per sample (RAF = AF/MSAF). MSAF for each sample is the allele frequency of the maximum allele among the single nucleotide variants for the particular sample.

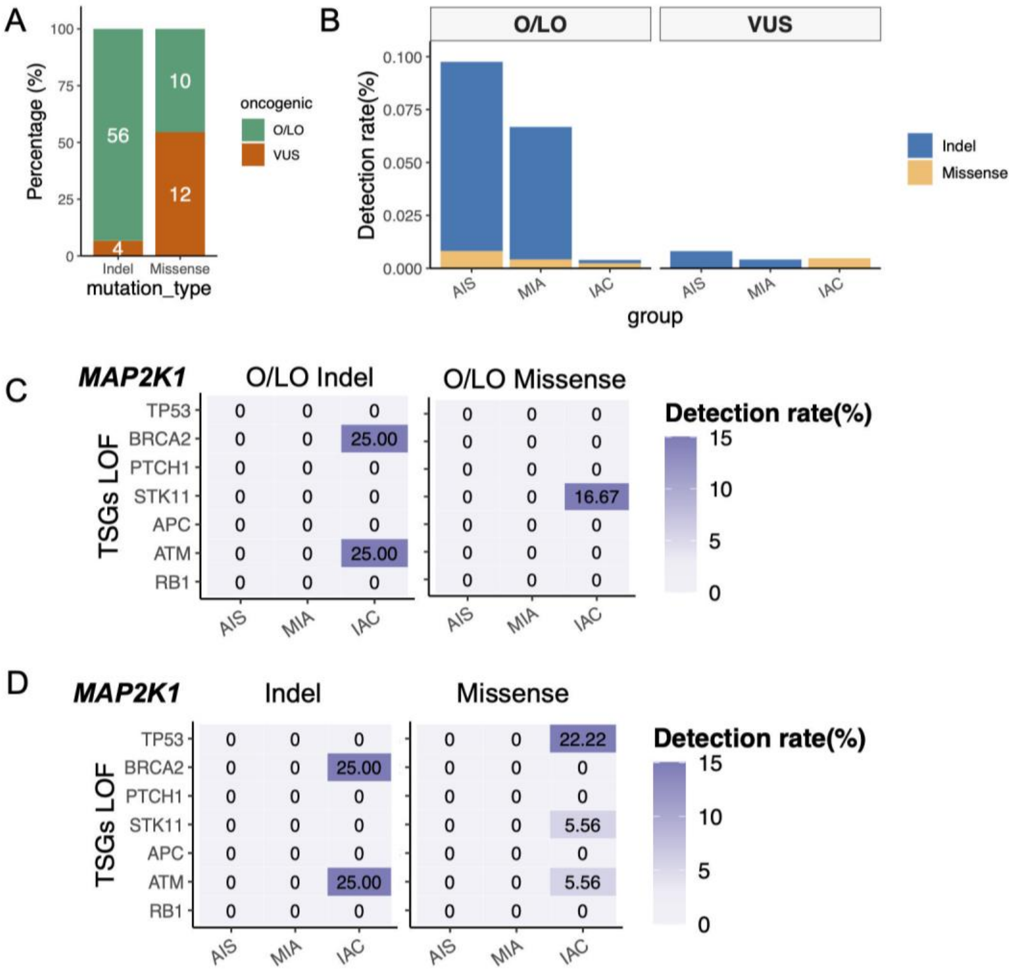

Figure S7. *MAP2K1* mutations were predominantly oncogenic/likely oncogenic. *MAP2K1* mutations were categorized using OncoKB database as oncogenic/likely oncogenic (O/LO) and variants of unknown significance (VUS). **A-B**. Component bar plots illustrating the distribution of O/LO and VUS mutations in all short insertion-deletion (Indel) and missense *MAP2K1* mutations (**A**) and across histological subtypes (**B**). **C-D.** Heat maps summarizing the concurrent detection rate of loss-of-function (LOF) mutations in various tumor suppressor genes (TSGs) in *MAP2K1* indels (left panel) and missense mutations (right panel) detected in only O/LO variants (**C**) and in the cohort (**D**) across histological subtypes. Zero (0) detection rate refers to the absence of detection of concurrent mutation in the indicated genes.

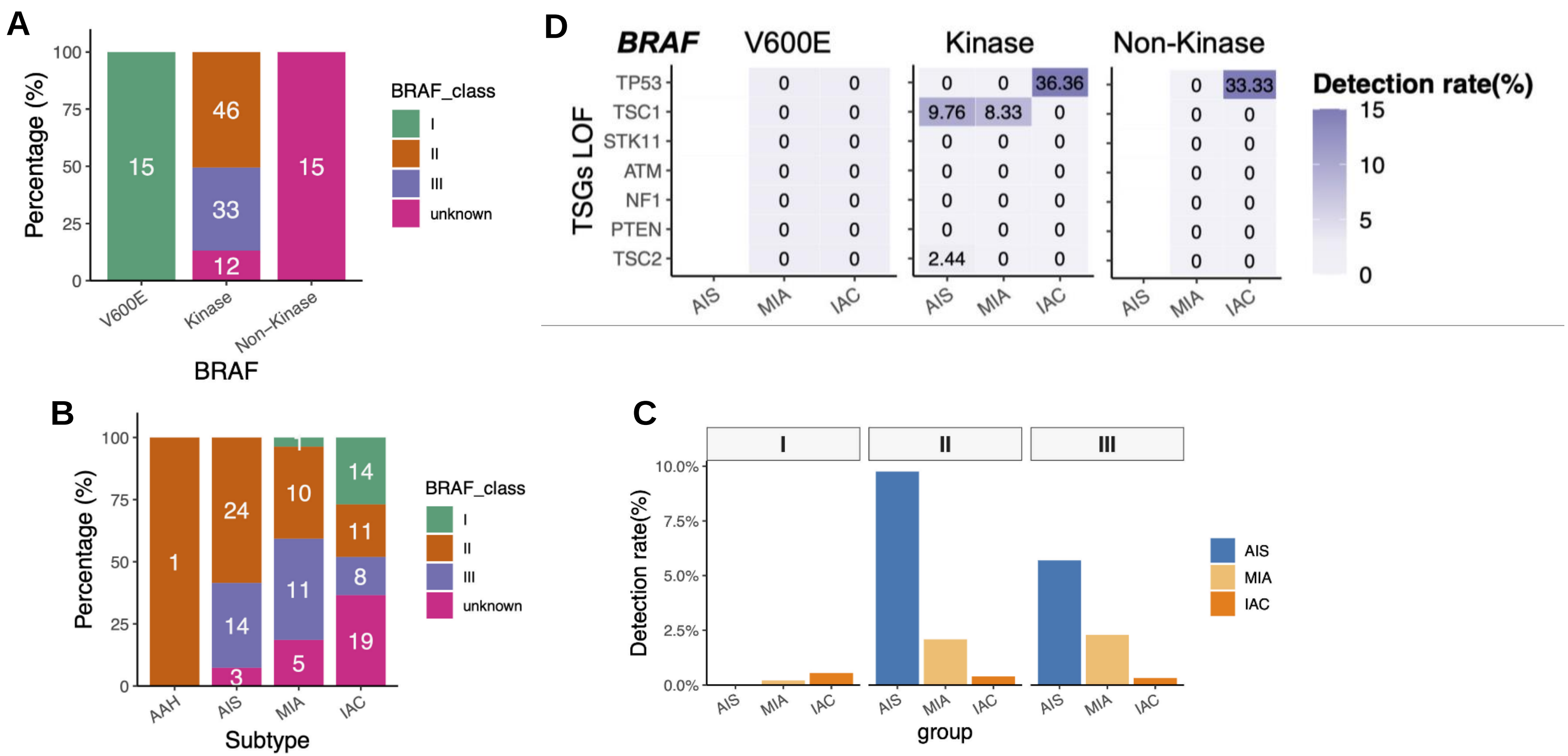

Figure S8. Distribution of *BRAF* mutations categorized according to kinase activity. *BRAF* mutations were categorized according to class I, II, and III. Class I, kinase-activating monomers, includes V600E/L. Class II, kinase-activating dimers, includes L597Q/R, G464V/A, G469A/V/R/S, K601E/N/T, E451Q, A712T, fusions. Class III, kinase-inactivating heterodimers includes G469E, G466V/E/A, N581S/I, D594G/N, G596R. **A-B**. Component bar plots illustrating the distribution of *BRAF* mutations according to class (**A**) and across histological subtypes (**B**). **C**. Distribution of *BRAF* mutation class I, II, and III in AIS, MIA, and IAC. **D**. Heat map summarizing the concurrent detection rate of loss-of-function (LOF) mutations in various tumor suppressor genes (TSGs) in *BRAF* V600E (left panel), kinase (middle panel), and non-kinase (right panel) across histological subtypes. Zero (0) detection rate refers to the absence of detection of concurrent mutation in the indicated genes. The blank columns or the absence of values refers to the absence of V600E and non-kinase mutations in AIS; hence, no data was available for concurrent mutation status.

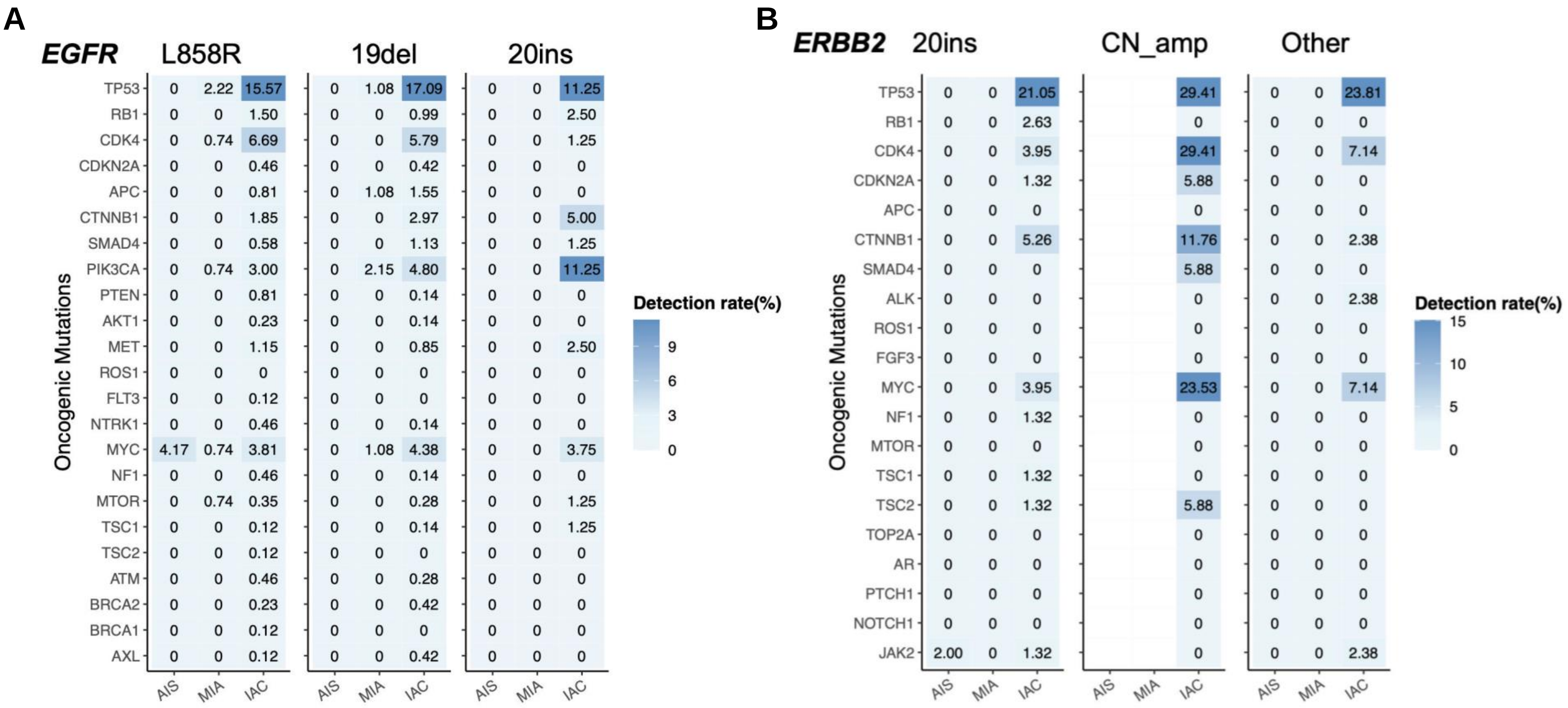

Figure S9. Concurrent mutation landscape in *EGFR*-mutant and *ERBB2*-mutant lung lesions. Heat maps summarizing concurrent detection rate of loss-of-function (LOF) mutations in various tumor suppressor genes (TSGs) and gain-of-function mutations in oncogenic genes in *EGFR*-mutant and *ERBB2*-mutant AIS, MIA, and IAC. Zero (0) detection rate refers to the absence of detection of concurrent mutation in the indicated genes. The blank columns or the absence of values refers to the absence of *ERBB2* copy number amplifications (CN amp) in AIS and MIA; hence, no data on concurrent mutations. Abbreviations: 19del, exon 19 deletion; 20ins, exon 20 insertion

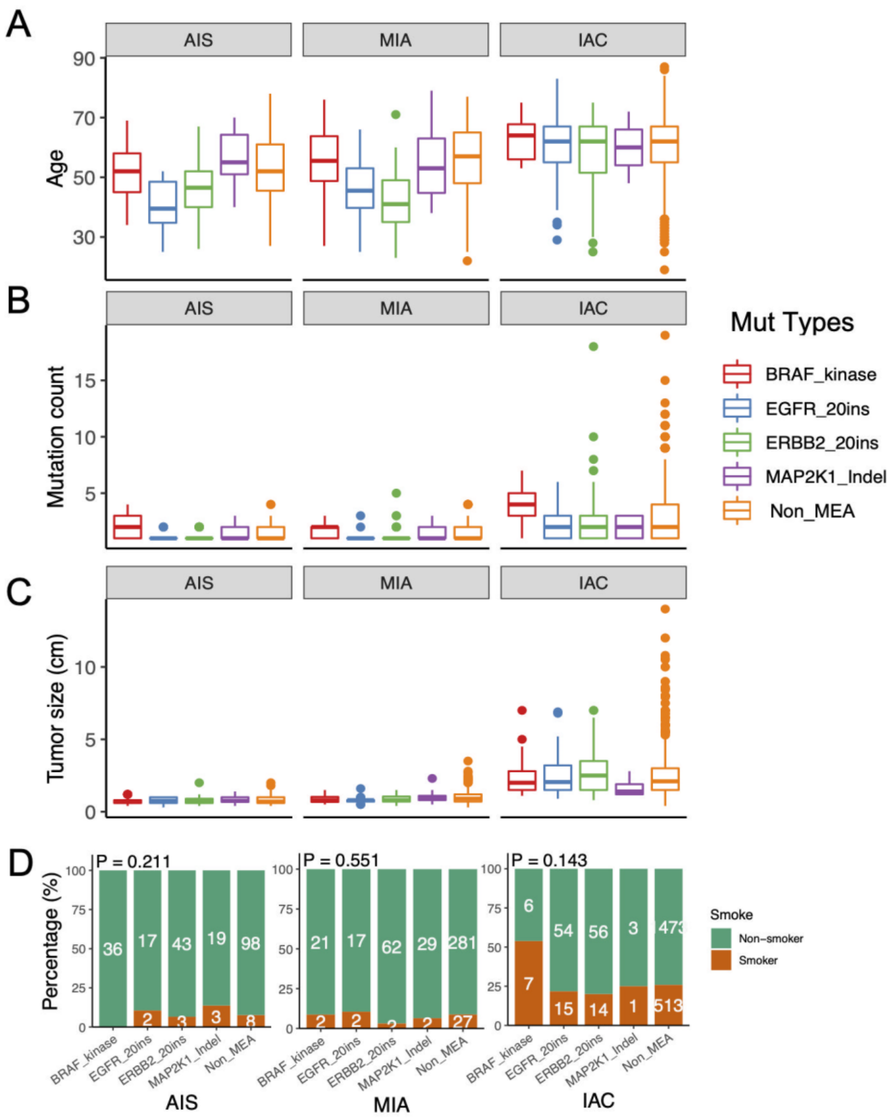

Figure S10. Distribution of age, mutation count, tumor size, and smoking history in adenocarcinoma in situ (AIS), minimally invasive adenocarcinoma (MIA), and invasive adenocarcinoma (IAC) according to mutations enriched in AIS (MEA) status. Box plots illustrating the distribution of age (**A**), mutation counts (**B**), tumor size (**C**), and smoking history (**D**) in AIS, MIA, and IAC harboring MEAs, including *BRAF* non-V600E located in kinase domain, *EGFR* exon 20 insertion (20ins), *ERBB2* 20ins, and *MAP2K1* small insertion-deletions (indel), and those without MEA (non-MEA).

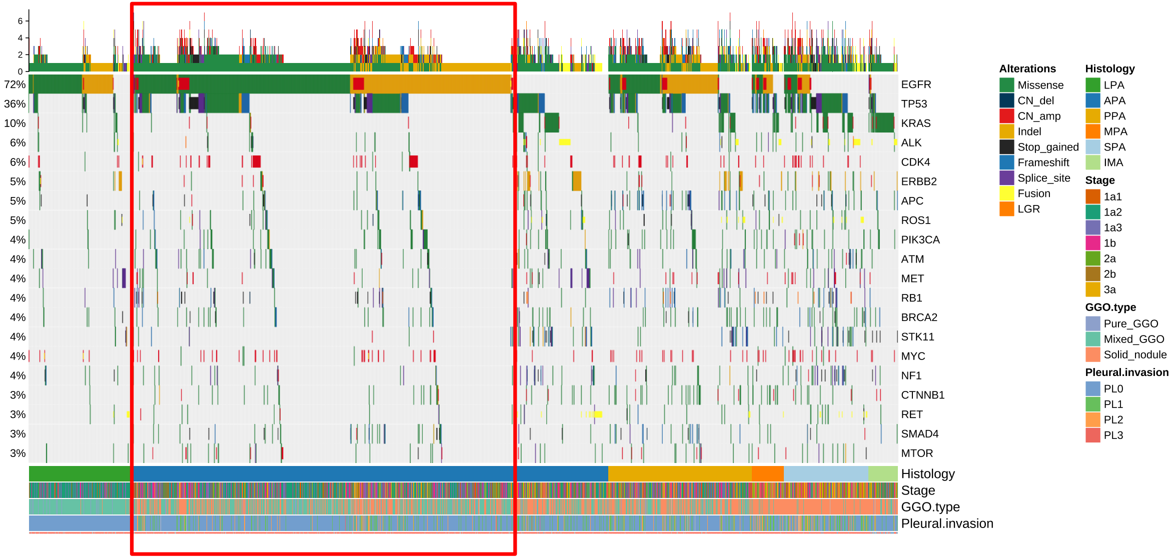

Figure S11. **Molecular features of IAC subtypes.** Oncoprint summarizing the mutation profile of the patients with invasive adenocarcinoma including lepidic-predominant adenocarcinoma (LPA), acinar-predominant adenocarcinoma (APA), papillary-predominant adenocarcinoma (PPA), micropapillary-predominant adenocarcinoma (MPA), solid-predominant adenocarcinoma (SPA), and invasive mucinous adenocarcinoma (IMA). Targeted sequencing was performed on surgically-resected tumor samples using a 68-gene panel. The histologic subtypes, pathological disease stage, pleural invasion status (PL), and radiological features (ground-glass opacity (GGO) type) of each patient are indicated by various colors at the bottom of the oncoprint. Each column represents a patient and each row represents a gene. Percentage on the left represents the percentage of patients with mutations in a specific gene indicated on the right. Top plot represents the overall number of mutations a patient harbored. Different colors denote the mutation types, including missense mutation, copy number deletion (CN_del), copy number amplification (CN_amp), insertion-deletion (indel), stop gained, frameshift, splice site variants, fusion, and large genomic rearrangements (LGR). Red frame indicates the *EGFR* mutation-enriched subset of APA.

Page 21

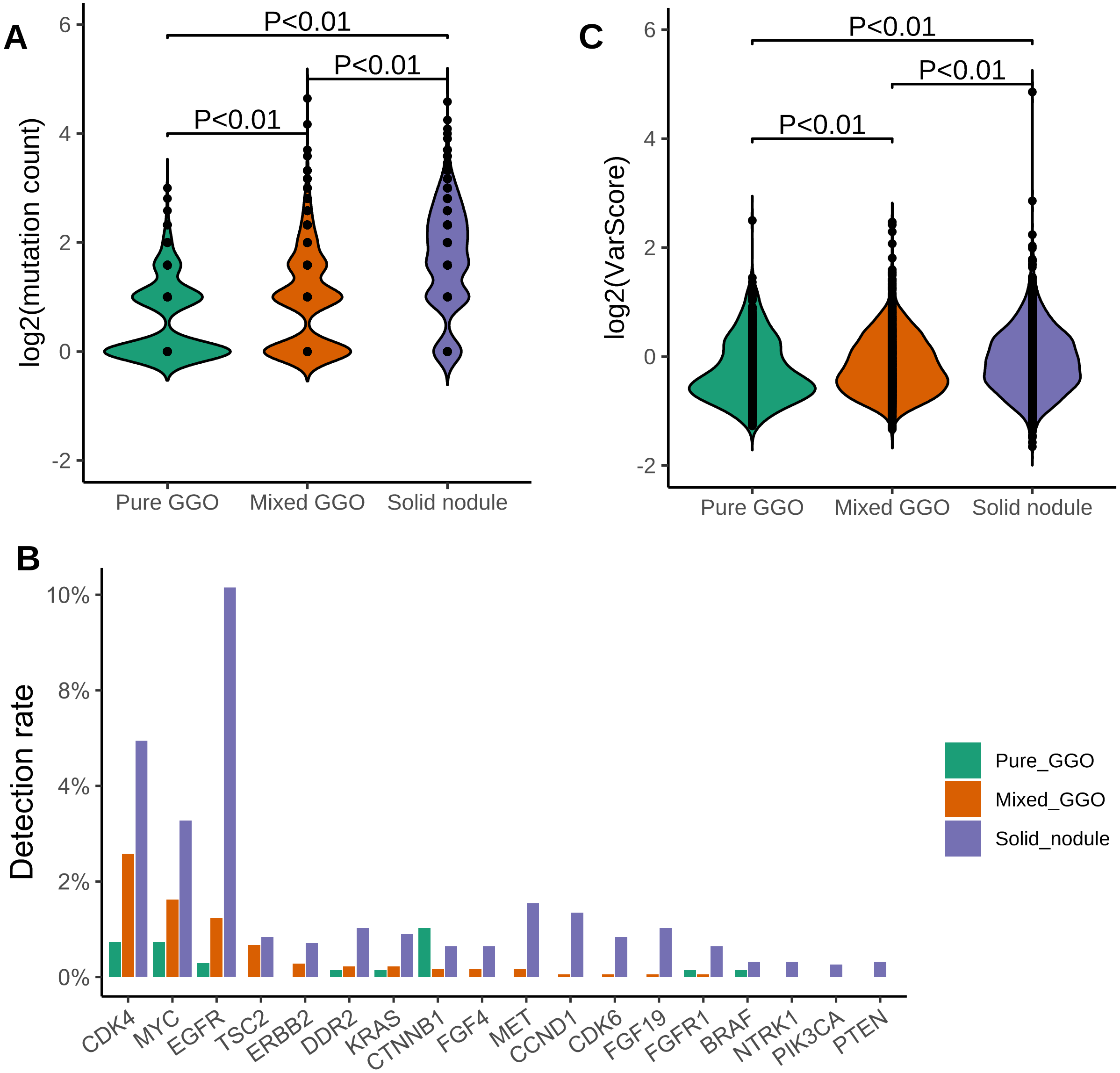

Figure S12. Molecular features according to radiological characteristics. A, C. Violin plots illustrating the distribution of mutation count (**A**) and copy number variations (CNV) varscore (**C**) of the cohort according to the three radiological features, pure ground-glass opacities (GGO), mixed GGO, and solid nodule. **B**. Histogram summarizing the distribution of CNVs detected from the cohort according to radiological features.

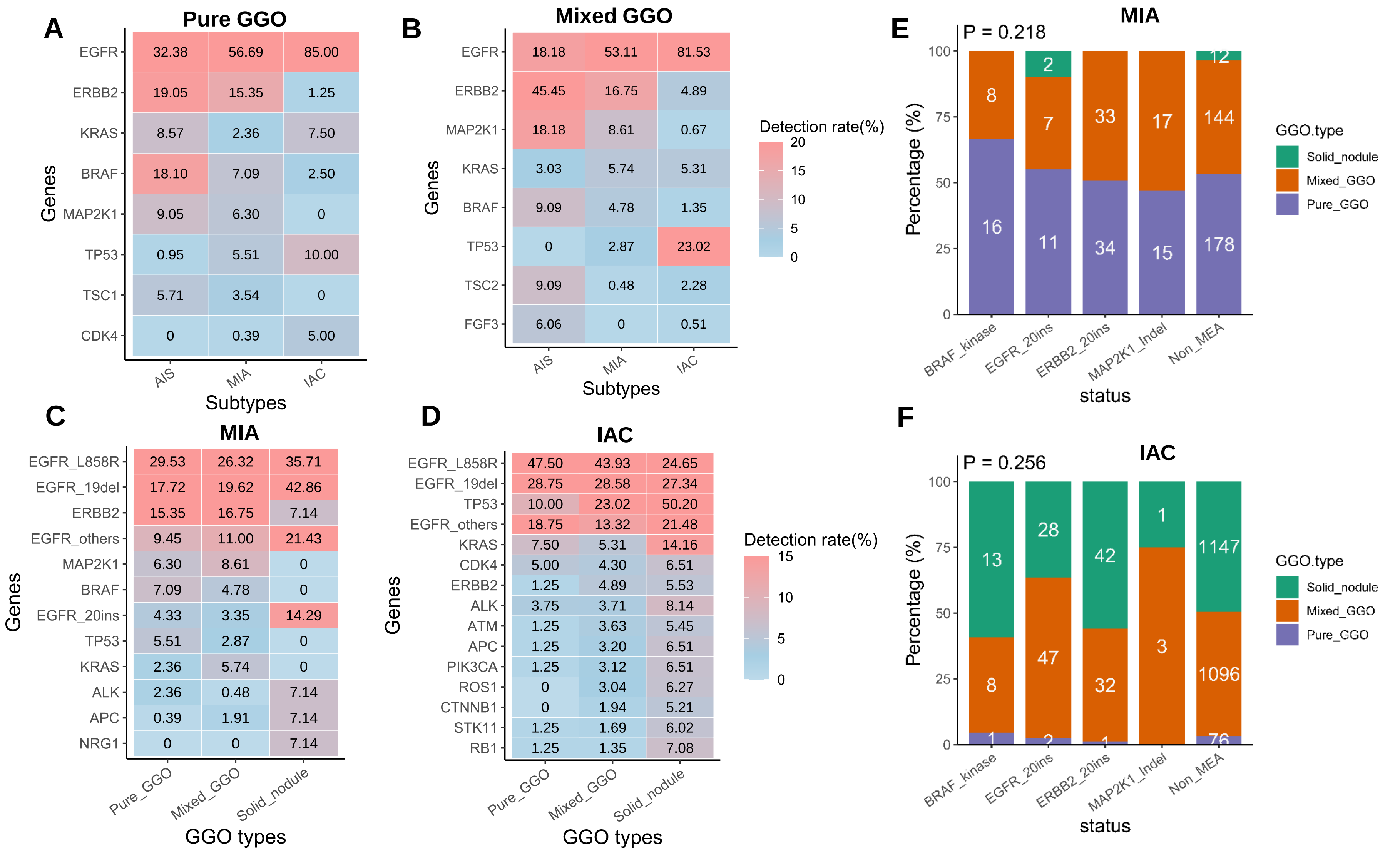

Figure S13. Pulmonary nodules characterized by either pure or mixed ground-glass opacities (GGO) have a similar mutation profile. A-B. Heat maps illustrating the differential rates of mutation detection in various genes according to: histological subtypes that appeared as pure GGO (**A**) or mixed GGO (**B**). **C-F**. Heat maps illustrating the distinct mutation profile (**C-D**) and component bar charts summarizing the distribution of mutations enriched in AIS (MEA) (**E-F**) across the three radiological features among minimally invasive adenocarcinoma (MIA) (**C,E**) and invasive adenocarcinoma (IAC) (**D, F**). Blue denotes a higher detection rate, while orange denotes a lower detection rate.
